# Hierarchical Nanoplatform Integrating Oxidative–Acidic Microenvironment Remodeling and Bone Homeostasis Modulation for Diabetic Bone Defect Therapy

**DOI:** 10.1002/advs.77841

**Published:** 2026-09-27

**Authors:** Xu Chen, Xingyu Zhu, Zhixin Qiu, Yijia Zhu, Xinhui Zheng, Dongqi Fan, Jinglei Zhan, Congxiu Mao, Ping Ji, Tao Chen, Qingqing He

**Affiliations:** ^1^ Chongqing Key Laboratory of Oral Diseases Chongqing Municipal Key Laboratory of Oral Biomedical Engineering of Higher Education Chongqing Municipal Health Commission Key Laboratory of Oral Biomedical Engineering The Affiliated Stomatological Hospital of Chongqing Medical University Chongqing China; ^2^ The Affiliated Yongchuan Hospital of Chongqing Medical University Chongqing Key Laboratory of Oral Diseases Chongqing Municipal Key Laboratory of Oral Biomedical Engineering of Higher Education Chongqing Municipal Health Commission Key Laboratory of Oral Biomedical Engineering The Affiliated Stomatological Hospital of Chongqing Medical University Chongqing China

**Keywords:** antioxidant nanozyme, diabetic bone defect repair, microenvironment regulation, mitochondrial homeostasis, osteogenesis‐osteoclastogenesis balance

## Abstract

Diabetic craniofacial bone defects exhibit unsatisfactory repair attributed to synergistic oxidative stress, local acidification and disordered bone immunity, whereas conventional mono‐functional biomaterials are incapable of synchronously resolving these interlocked pathological cascades. Herein, a hierarchical nanoplatform (CM/LDH) is fabricated by immobilizing Ce‐MOF nanozyme onto CaAl‐LDH substrate for synergistic bone remediation. As an alkaline and bioactive support, CaAl‐LDH improves peri‐defect microenvironment acidification to preserve the redox catalytic activity of loaded Ce‐MOF, accompanied with sustained Ca^2^^+^ release to support BMSC viability and osteogenic differentiation. The immobilized Ce‐MOF component eliminates excessive ROS via reversible Ce^3^^+^/Ce^4^^+^ cycling, further alleviating inflammatory polarization of macrophages and cutting off pathological cell crosstalk. Benefiting from mutual complementary functions, CM/LDH synchronously rectifies oxidative‐acidic microenvironment and rebalances osteogenic‐osteoclastic turnover, overcoming inherent drawbacks of single‐target interventions. This synergistic nanosystem provides a potential biomaterial option for diabetic craniofacial bone regeneration.

## Graphical abstract

A hierarchical CM/LDH nanoplatform integrates ROS‐scavenging Ce‐MOF nanozymes with acid‐neutralizing CaAl‐LDH and is delivered within GelMA hydrogel to diabetic bone defects. By remodeling the oxidative‐acidic microenvironment, preserving mitochondrial homeostasis, and suppressing mtDNA–cGAS–STING inflammatory signaling, CM/LDH reduces proinflammatory macrophage activation, thereby restoring osteoimmune homeostasis, rebalancing osteoblast–osteoclast activity, and promoting bone regeneration.

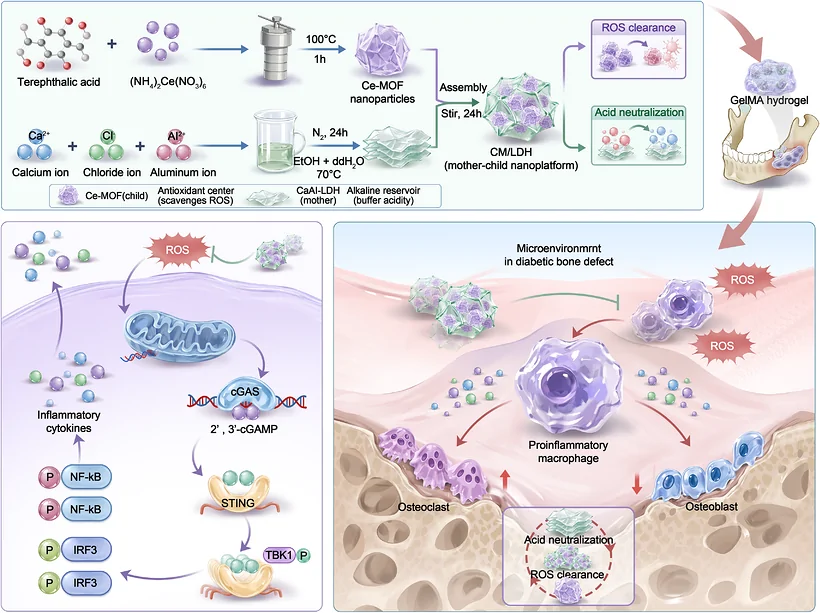

## 1 Introduction

Diabetic bone defects represent a challenging form of skeletal injury in which local tissue repair is profoundly affected by systemic metabolic dysregulation [1, 2]. Compared with non‐diabetic individuals, patients with diabetes exhibit a higher fracture risk, prolonged healing time, and an increased likelihood of delayed or incomplete bone repair [3, 4]. These unfavorable regenerative outcomes are closely associated with the diabetic pathological microenvironment, which is characterized by persistent hyperglycemia, excessive reactive oxygen species (ROS) accumulation, chronic inflammation, and dysfunction of regeneration‐related cells [5, 6, 7, 8]. Craniofacial bone is a highly dynamic tissue whose structural integrity depends on the coordinated balance between osteoblast‐mediated bone formation and osteoclast‐mediated bone resorption [9, 10]. Under diabetic conditions, however, this balance is readily disrupted, leading to uncoupled bone remodeling and delayed defect healing [11, 12]. Therefore, regenerative strategies for diabetic craniofacial bone defects should not only support bone formation, but also modulate the specific pathological features of the diabetic microenvironment.

In the complex pathological context of diabetic chronic inflammation complicated by acute inflammatory bone damage, hyperglycemia‐driven reactive oxygen species (ROS) accumulation is not an independent event. Instead, it acts synergistically with inflammatory metabolic byproducts such as lactic acid to establish an oxidative‐acidic microenvironment that impedes bone regeneration [13, 14]. Previous studies have shown that pathological bone defects associated with diabetes or chronic inflammation usually exhibit a weakly acidic extracellular microenvironment, with the bulk pH ranging from 6.0 to 6.8 [14, 15]. Furthermore, excessive ROS potentiates osteoclast‐associated signaling pathways mediated by the RANKL/RANK/OPG axis, leading to robust osteoclast hyperactivation [16]. These overactivated osteoclasts release substantial hydrogen ions (H^+^) into the defective microenvironment, where the resultant local acidification directly accelerates bone mineral dissolution and markedly inhibits osteoblastic function [17, 18]. Collectively, ROS‐mediated oxidative damage superimposes on acidic products‐induced acid etching of bone tissue, thereby constructing a self‐perpetuating pathological microenvironment that resists spontaneous reversal and repair. Moreover, this adverse physicochemical environment promotes macrophage polarization toward a proinflammatory phenotype, resulting in increased secretion of inflammatory cytokines such as IL‐6 and TNF‐α [19, 20, 21]. At the intracellular level, macrophage mitochondrial homeostasis is highly vulnerable to diabetic oxidative stress; excessive ROS can induce mitochondrial damage, mitochondrial fragmentation, and mitochondrial DNA leakage, thereby activating innate immune inflammatory pathways such as the cGAS‐STING/NF‐κB axis [22, 23]. These mitochondria‐associated inflammatory signals further aggravate osteoblast injury, osteoclast activation, and bone marrow mesenchymal stem cell dysfunction or senescence, thereby sustaining pathological crosstalk among immune cells, osteoblasts, and osteoclasts [24, 25, 26]. Thus, diabetic bone defects are not driven by a single pathological factor, but by a mutually reinforcing cycle of oxidative stress, local acidification, and osteoimmune dysregulation. This complexity may explain why isolated antioxidant, antiresorptive, anti‐inflammatory, or osteogenic interventions often fail to sufficiently reconstruct a regenerative microenvironment [19].

Antioxidant nanozymes provide a materials‐based strategy for regulating excessive oxidative stress in diabetic bone defects [20, 27, 28]. Among them, cerium‐based nanozymes can scavenge ROS through reversible redox cycling between Ce^3^^+^ and Ce^4^^+^, thereby mimicking enzyme‐like activities such as superoxide dismutase and catalase [29]. Cerium‐based metal–organic frameworks (Ce‐MOFs) are particularly attractive because their porous and designable structures can provide abundant catalytic sites and tunable biological functions [30]. However, the antioxidant performance of cerium‐based nanozymes is highly dependent on the surrounding microenvironment. Under acidic pathological conditions, excessive H^+^ may interfere with the conversion of Ce^4^^+^ to Ce^3^^+^, thereby weakening the sustained redox cycling required for ROS clearance [31, 32]. More importantly, ROS scavenging alone cannot fully resolve osteoblast–osteoclast imbalance or inflammatory osteoimmune crosstalk in diabetic bone defects. Therefore, an effective material system should not only provide a favorable physicochemical environment for Ce‐MOF catalysis, but also simultaneously correct local acidification and bone‐remodeling imbalance.

Layered double hydroxides (LDHs) provide a complementary platform for addressing these challenges because of their favorable biocompatibility, pH‐responsive degradation, acid‐neutralizing capacity, and delivery potential [33, 34]. Among various LDH formulations, CaAl‐LDH is particularly suitable for diabetic bone regeneration because it combines mild acid buffering with degradation‐mediated Ca^2^^+^ release [35]. Compared with MgAl‐LDH or other non‐calcium LDHs, CaAl‐LDH provides a direct osteogenesis‐relevant ionic cue through Ca^2^^+^ supplementation [36, 37, 38]. Therefore, CaAl‐LDH is not merely a passive carrier, but an active microenvironment‐regulating component capable of supporting acid neutralization, Ce‐MOF stabilization, and osteogenic recovery. By neutralizing local acidity, CaAl‐LDH may improve the catalytic conditions required for Ce‐MOF‐mediated ROS clearance; by reducing acid‐associated osteoclast activity and releasing osteogenesis‐supportive Ca^2^^+^, it may further contribute to the restoration of osteoblast–osteoclast balance. These properties suggest that integrating Ce‐MOF with CaAl‐LDH could provide a coordinated intervention strategy targeting the coupled oxidative and acidic pathology of diabetic bone defects.

**SCHEME 1:**
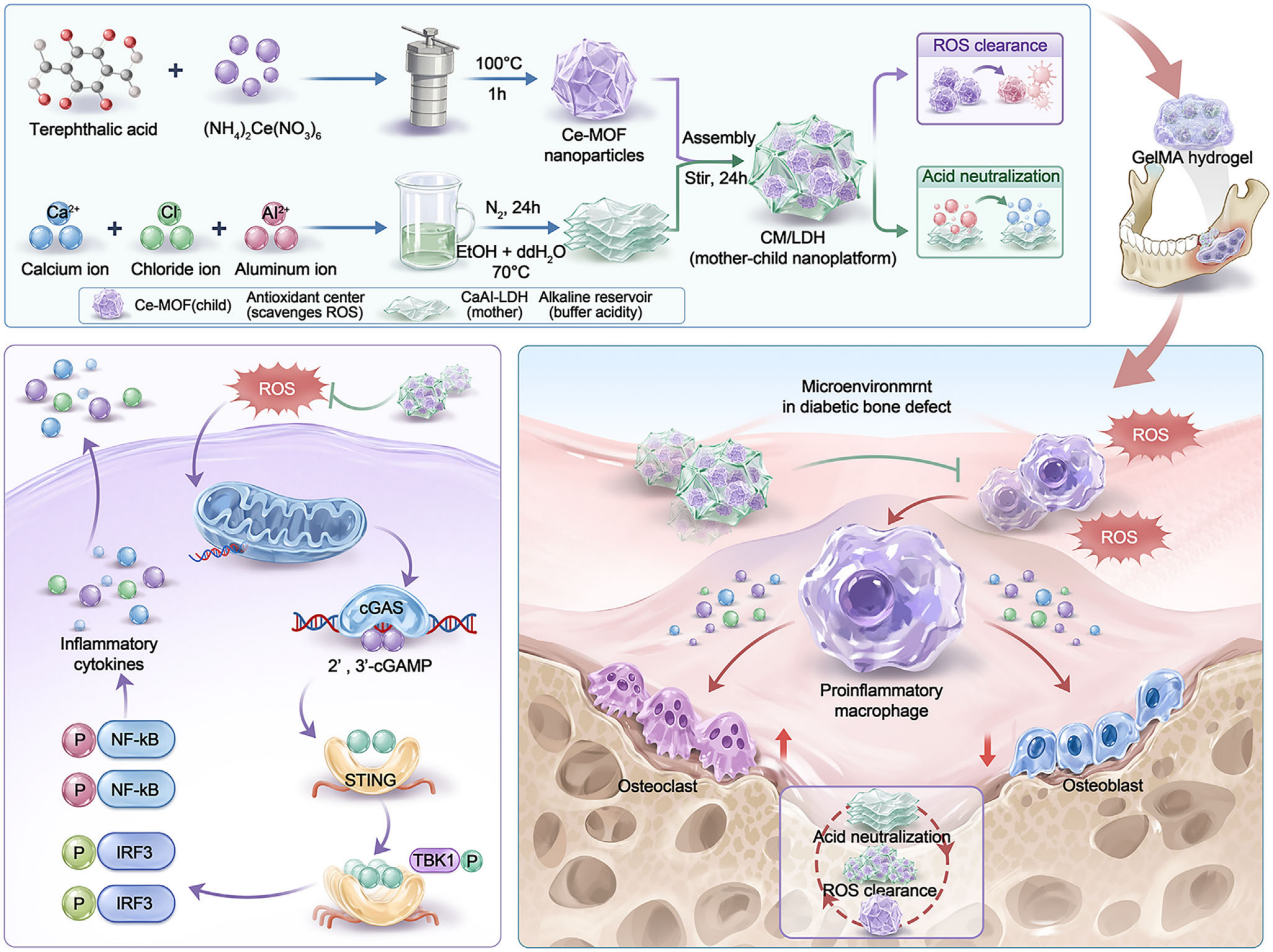
Schematic illustration of a mother‐child nanoplatform for bone microenvironment remodeling and regulation of osteoblast‐osteoclast balance. First, cerium‐based metal‐organic frameworks (Ce‐MOF) were synthesized via a hydrothermal reaction of ammonium cerium nitrate and terephthalic acid in a reaction kettle. Calcium‐aluminum layered double hydroxides (CaAl‐LDH) were prepared through an organic/aqueous solution route. Finally, the CM/LDH mother‐child nanoplatform was constructed by loading Ce‐MOF into CaAl‐LDH. The child drone Ce‐MOF with reactive oxygen species (ROS) scavenging capacity and the mother carrier CaAl‐LDH with acid neutralization capability exert synergistic functions. After the CM/LDH nanoplatform is delivered to diabetic bone defect sites, the ROS‐dominated diabetic microenvironment is substantially alleviated, the inflammatory phenotype of macrophages is effectively modulated, and the imbalance between osteogenesis and osteoclastogenesis is ultimately restored. Further mechanistic studies reveal that CM/LDH eliminates excessive ROS, maintains mitochondrial homeostasis in macrophages, reduces mitochondrial DNA (mtDNA) leakage, inhibits the activation of the cGAS‐STING pathway, and consequently downregulates the expression of pro‐inflammatory cytokines in macrophages. This work provides a synergistic intervention strategy to rebalance osteogenesis and osteoclastogenesis for the repair of diabetic craniomaxillofacial bone defects.

Based on these considerations, a CaAl‐LDH‐based mother‐child nanoplatform loaded with Ce‐MOF nanoparticles, termed CM/LDH, was constructed to coordinately regulate the oxidative–acidic microenvironment, osteoblast–osteoclast imbalance and osteoimmune dysregulation in diabetic bone defects (Scheme 1). Unlike conventional strategies based on single antioxidant intervention or isolated osteogenic induction, the CM/LDH nanoplatform was designed based on a proposed functional model in which CaAl‐LDH‐mediated acid buffering creates a more favorable microenvironment that may support Ce‐MOF‐mediated ROS scavenging and subsequent cellular regulation. In this hybrid system, CaAl‐LDH functions as an alkaline and bioactive support that buffers local acidity, provides Ca^2^^+^‐associated osteogenic cues, and helps maintain a favorable physicochemical environment for Ce‐MOF catalysis. Ce‐MOF serves as the ROS‐scavenging nanozyme component that alleviates oxidative stress and macrophage‐associated inflammatory activation. We further hypothesized that this ROS‐scavenging and acid‐buffering strategy could preserve macrophage mitochondrial homeostasis and suppress mtDNA‐mediated cGAS‐STING inflammatory signaling, thereby interrupting macrophage‐driven pathological intercellular communication. This mutually reinforcing design enables CM/LDH to achieve tissue regeneration in the multifactorial pathological setting of diabetic bone defects, a task that is difficult to accomplish with single‐component systems, and provides a referential materials design strategy for diabetic craniofacial bone repair.

## 2 Results and Discussion

### 2.1 Construction of the CM/LDH Mother‐Child Nanoplatform With Acid‐Buffered ROS‐Scavenging Activity

**FIGURE 1:**
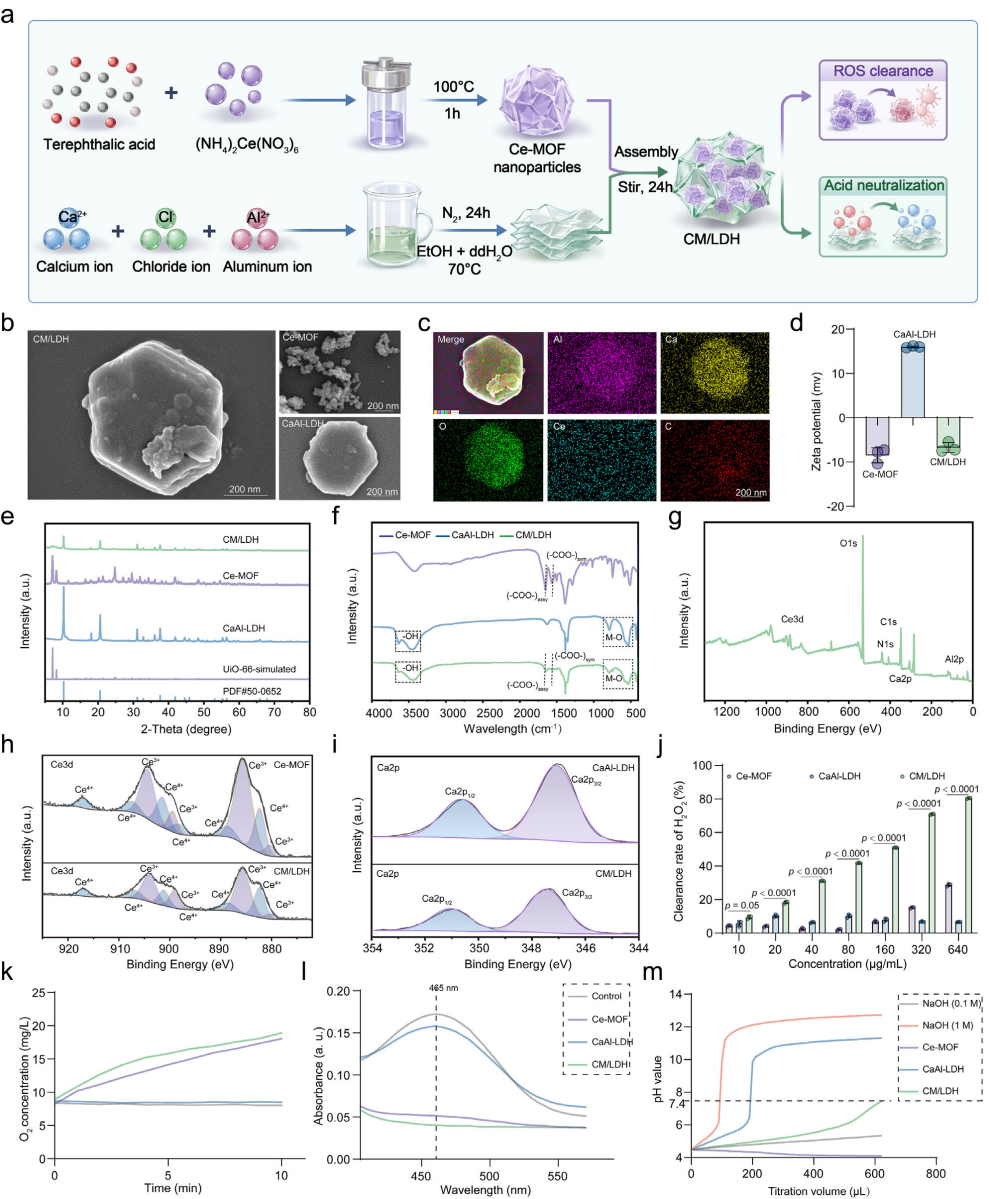
Synthesis and characterization of the CM/LDH mother–child nanoplatform. (a) Schematic diagram of the synthesis process for the CM/LDH nanoplatform. (b) Scanning electron microscopy images of CM/LDH, Ce‐MOF, and CaAl‐LDH. (c) High‐angle annular dark‐field (HAADF) and elemental mapping images of CM/LDH. (d) Zeta potential of Ce‐MOF, CaAl‐LDH and CM/LDH. (e) XRD patterns of Ce‐MOF, CaAl‐LDH and CM/LDH. (f) FTIR spectra of Ce‐MOF, CaAl‐LDH and CM/LDH. (g) XPS survey spectrum of CM/LDH. (h) High‐resolution XPS spectra showing elemental analysis of cerium in Ce‐MOF and CM/LDH. (i) High‐resolution XPS spectra showing elemental analysis of Calcium in CaAl‐LDH and CM/LDH. (j) The H_2_O_2_ clearance rate from materials at different concentrations. (k) O_2_ generation curves under different treatments. (l) Absorbance at 465 nm for quantifying •O_2_^−^ scavenging capacity of various materials using the WST‑8‑based superoxide dismutase assay. (m) pH value monitoring during titration of different samples into acidic PBS (pH 4.5). Data in bar plots are presented as the mean ± SD. n ≥ 3 independent samples. Statistical significance was determined by one‐way ANOVA.

The CM/LDH mother‐child nanoplatform was constructed by assembling Ce‐MOF nanoparticles onto CaAl‐LDH nanosheets, as shown in Figure 1a. Scanning electron microscope (SEM) images demonstrated that CaAl‐LDH presented a typical sheet‐like morphology with a lateral size of approximately 500 nm, whereas Ce‐MOF appeared as nanoparticles with an average size of approximately 20 nm. After assembly, CM/LDH retained the morphological features of both components, with Ce‐MOF nanoparticles distributed on the CaAl‐LDH nanosheets (Figure 1b). HAADF‐STEM and elemental mapping further revealed the coexistence of Ca and Al signals from CaAl‐LDH and Ce and C signals from Ce‐MOF within the hybrid structure, supporting the successful loading of Ce‐MOF onto the LDH carrier (Figure 1c and Figure S1). Zeta potential measurements showed that Ce‐MOF and CaAl‐LDH exhibited surface potentials of approximately −8 and 16 mV, respectively, while CM/LDH displayed an intermediate potential of approximately −6 mV (Figure 1d). This potential shift suggests that electrostatic attraction contributes to the assembly between the negatively charged Ce‐MOF nanoparticles and positively charged CaAl‐LDH nanosheets. Dynamic light‑scattering (DLS) measurements revealed that the hydrodynamic size of Ce‑MOF was approximately 20 nm, whereas both CaAl‑LDH and CM/LDH exhibited hydrodynamic sizes ranging from 400 to 500 nm (Figure S2). Thermogravimetric analysis (TGA) was also conducted to roughly evaluate the loading amount of Ce‐MOF on CaAl‐LDH in CM/LDH. As presented in Figure S3, the composition of CM/LDH was estimated using a linear mixing model in the relatively comparable temperature region of 500°C–650°C. The results suggest that CM/LDH contains approximately 11.9 wt.% Ce‐MOF and 88.1 wt.% CaAl‐LDH, corresponding to a Ce‐MOF/CaAl‐LDH mass ratio of about 1:7.4.

The crystalline structure and chemical coupling of CM/LDH were further investigated by x‐ray diffraction (XRD), FTIR, and x‐ray photoelectron spectroscopy (XPS). XRD patterns showed the characteristic diffraction peaks of layered CaAl‐LDH, while Ce‐MOF displayed diffraction signals corresponding to UiO‐66(Ce)‐type frameworks. CM/LDH retained the representative peaks of both components, indicating that the crystalline features of Ce‐MOF and CaAl‐LDH were largely preserved after assembly (Figure 1e). FTIR spectra further supported the coexistence of the two components. Ce‐MOF exhibited characteristic carboxylate vibrations at 1641–1554 and 1435—1373 cm^−^^1^, corresponding to asymmetric and symmetric stretching of coordinated ─COO^−^ groups, whereas CaAl‐LDH showed broad ─OH stretching at 3630–3475 cm^−^^1^ and metal ─OH vibrations at 788 and 538 cm^−^^1^. The CM/LDH spectrum inherited the main absorption features of both Ce‐MOF and CaAl‐LDH, supporting the formation of the MOF–LDH composite (Figure 1f). XPS analysis provided additional evidence for interfacial coupling. The CM/LDH survey spectrum contained the characteristic elemental signals of both components, and high‐resolution spectra showed binding‐energy shifts and peak redistribution in C 1s, O 1s, Ce 3d, Ca 2p, and Al 2p regions compared with the individual materials (Figures 1g–i and S4). These changes suggest interfacial electronic communication between Ce‐MOF and CaAl‐LDH within the hybrid nanostructure. These structural and chemical characterization results indicate that CM/LDH represents a hierarchical MOF‐on‐LDH hybrid structure, in which Ce‐MOF nanoparticles are immobilized on CaAl‐LDH nanosheets rather than simply mixed with the LDH component.

In the H_2_O_2_ decomposition assay, CaAl‐LDH showed limited catalytic activity, whereas Ce‐MOF exhibited concentration‐dependent H_2_O_2_ scavenging. Notably, CM/LDH showed a markedly enhanced H_2_O_2_ clearance efficiency, reaching nearly 80% at the tested concentration (Figure 1j). Consistently, dissolved oxygen generation assays demonstrated that CM/LDH promoted more efficient H_2_O_2_ decomposition than Ce‐MOF alone (Figure 1k). Superoxide anion radical (·O_2_^−^) scavenging was further assessed using a WST‐8‐based assay. Both Ce‐MOF and CM/LDH showed evident ·O_2_^−^ scavenging activity, indicating that the ROS‐scavenging capability of Ce‐MOF was retained after assembly with CaAl‐LDH (Figure 1l). Together, these results suggest that CM/LDH not only preserves the antioxidant activity of Ce‐MOF but may also benefit from the MOF–LDH heterointerface, which could facilitate electron transfer during H_2_O_2_ decomposition.

Because diabetic bone defects are accompanied by local acidification and excessive H^+^ may impair the Ce^3^^+^/Ce^4^^+^ redox cycling of Ce‐MOF, the acid‐buffering behavior of CM/LDH was further examined. Titration experiments in acidic PBS showed that Ce‐MOF slightly decreased the solution pH with increasing dosage, whereas CaAl‐LDH displayed strong acid‐neutralizing capacity. Compared with CaAl‐LDH alone, CM/LDH produced a milder and more gradual pH increase, suggesting a controlled buffering effect derived from the LDH component (Figure 1m). The ROS‐scavenging activity of these materials was also evaluated under different pH conditions. Ce‐MOF showed moderate H_2_O_2_ degradation at pH 7.4, but its activity was substantially weakened under acidic conditions at pH 5.0. In contrast, CM/LDH maintained effective H_2_O_2_ degradation at both pH 7.4 and pH 5.0, outperforming Ce‐MOF and CaAl‐LDH alone (Figure S5). To clarify the interaction between CaAl‐LDH and Ce‐MOF in CM/LDH and the origin of its enhanced antioxidant activity, XPS was used to analyze the Ce^3^^+^/Ce^4^^+^ ratio under different conditions (Figure S6). Since Ce^3^^+^ sites are closely related to oxygen vacancies and ROS scavenging, this ratio is an important indicator of antioxidant activity. Physical mixing of Ce‐MOF with CaAl‐LDH showed a Ce^3^^+^/Ce^4^^+^ ratio similar to that of Ce‐MOF but much lower than that of CM/LDH, indicating strong interfacial coupling rather than simple blending. After reaction with H_2_O_2_, CM/LDH exhibited a larger decrease in the Ce^3^^+^/Ce^4^^+^ ratio than Ce‐MOF, suggesting more active participation of Ce^3^^+^ sites in H_2_O_2_ reduction and ROS elimination. Under acidic conditions (pH 5.0), the Ce^3^^+^/Ce^4^^+^ ratio of Ce‐MOF decreased markedly compared with that at pH 7.4, indicating reduced stability of Ce^3^^+^ active sites. In contrast, CM/LDH maintained a relatively stable Ce^3^^+^/Ce^4^^+^ ratio, suggesting that CaAl‐LDH buffers the acidic microenvironment and stabilizes the Ce^3^^+^/Ce^4^^+^ redox cycle. Further electrochemical impedance spectroscopy (EIS) and cyclic voltammetry (CV) demonstrated that CM/LDH exhibits lower charge‐transfer resistance, higher peak currents and broader electrochemical response windows compared with Ce‐MOF (Figure S7). This indicates improved electrocatalytic activity and electron‐transfer capability. Overall, the superior antioxidant performance of CM/LDH originates from interfacial electronic interactions between Ce‐MOF and CaAl‐LDH, a stable Ce^3^^+^/Ce^4^^+^ redox cycle, and acid‐neutralization by CaAl‐LDH, enabling efficient and sustained ROS scavenging in acidic pathological microenvironments.

The acid‐responsive release profiles of Ca and Ce ions from CM/LDH over time were further monitored by ICP‐OES (Figure S8). Compared with neutral conditions, the release rates of both ions were markedly enhanced in the acidic solution, indicating the pH‐responsive behavior of CM/LDH. And it can be observed from the results that both Ca and Ce ions are released at biocompatible concentrations [39, 40]. However, Ca and Ce ions exhibited distinct time‐dependent release patterns. Ca ions were released at a much faster rate and reached a plateau earlier, whereas Ce ions displayed a slower and more sustained release profile. These results suggest that the CaAl‐LDH component in CM/LDH can rapidly respond to and degrade in an acidic microenvironment, thereby contributing to the amelioration of local acidity. This rapid response may further provide favorable conditions for the slower and prolonged release of Ce ions, enabling their sustained antioxidant activity. These findings indicate that CaAl‐LDH serves not only as a carrier matrix but also as an acid‐buffering component that may help preserve Ce‐MOF‐associated antioxidant activity under acidic pathological conditions. We also quantified aluminum release from CM/LDH under physiological and acidic conditions. Following 3‑day incubation at pH 7.4, the aluminum concentration reached a trace level of ∼0.121 mg L^−^^1^ and slightly rose to 5.967 mg L^−^^1^ under acidic conditions (Table S1). Therefore, degradation‑associated aluminum release from the material is limited and far below the concentration threshold for inducing overt cytotoxicity [41].

### 2.2 CM/LDH Regulates the Fate of Osteoclasts via Acid Neutralization

Before evaluating the regulatory effect of CM/LDH on osteoclasts, the cytocompatibility of the synthesized nanoparticles was evaluated in bone marrow mesenchymal stem cells (BMSCs). CCK‐8 assays showed that Ce‐MOF, CaAl‐LDH, and CM/LDH caused no obvious cytotoxicity at concentrations below 1000 µg mL^−^^1^ (Figure S9a). Notably, the CM/LDH group slightly promoted BMSCs proliferation at concentrations ranging from 125 to 500 µg mL^−^^1^. Consistently, Calcein/PI live/dead staining showed almost no PI‐positive cells after treatment with the nanomaterials at 250 µg mL^−^^1^ (Figure S9b). Based on these results, 250 µg mL^−^^1^ was chosen as the working concentration for subsequent cell experiments.

**FIGURE 2:**
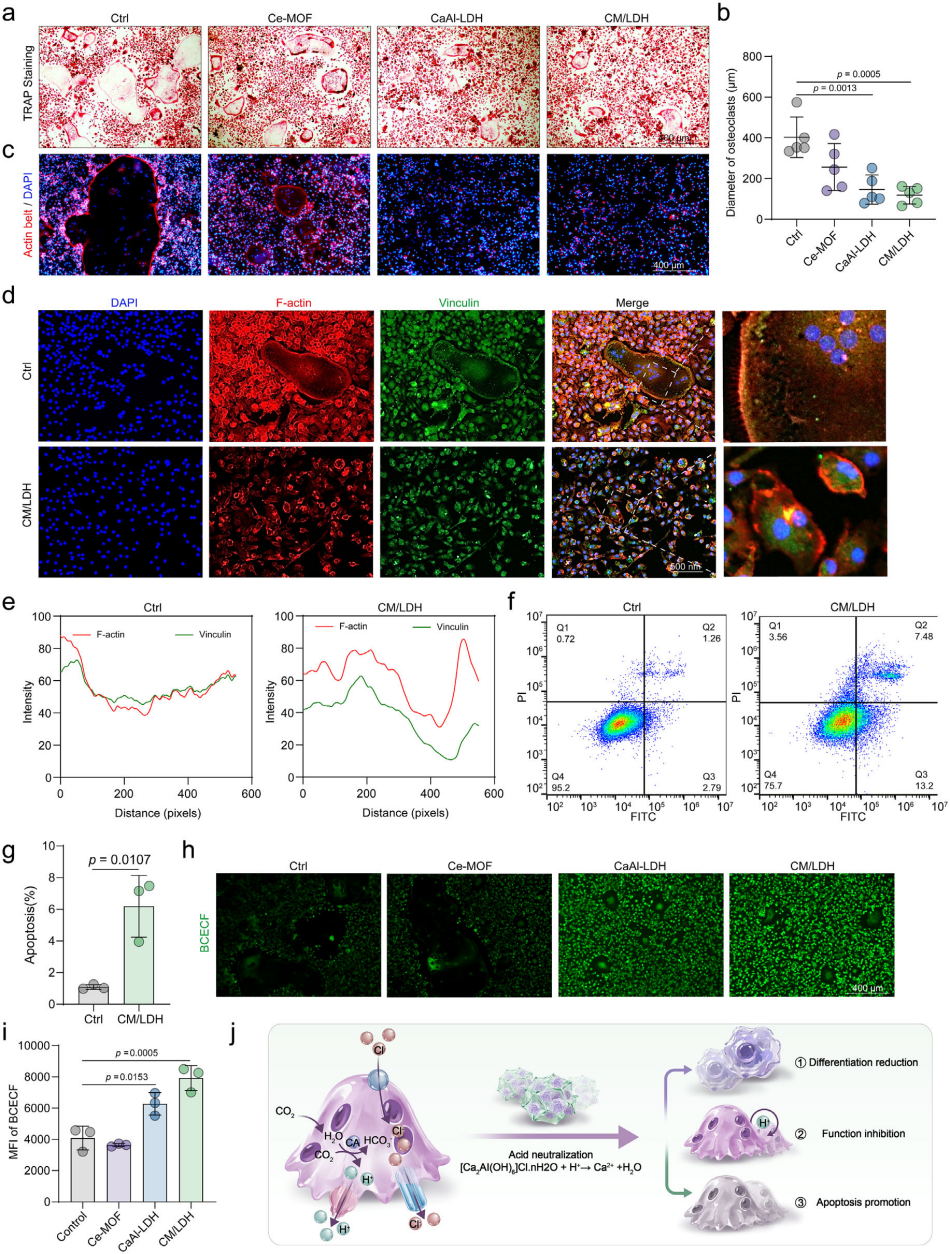
CM/LDH regulates osteoclast fate through acid neutralization. (a,b) Representative TRAP staining images and quantitative statistics of osteoclast with different treatments. (c) Representative cytoskeleton fluorescence images of osteoclast with different treatments. (d) Representative immunofluorescence images of osteoclast co‐stained with anti‐vinculin antibody (green), rhodamine phalloidin for F‐actin (red), and DAPI (blue). (e) Line‐scanning profiles of green and red fluorescence in selected white dashed box region indicated in (d). (f,g) Flow cytometry analysis and quantification of osteoclast apoptosis after CM/LDH treatment using Annexin V‐FITC/PI staining. (h,i) Representative BCECF‐AM fluorescence images and quantitative analysis of relative intracellular alkalinization in osteoclasts after different treatments. (j) Schematic diagram of CM/LDH regulating osteoclast fate through acid neutralization. Data in bar plots are presented as the mean ± SD. n ≥ 3 biologically independent samples. Statistical significance was determined by one‐way ANOVA.

Given the important contribution of excessive osteoclast activity to diabetic bone loss and delayed defect repair, the direct effect of CM/LDH on osteoclastogenesis was first examined. Tartrate‐resistant acid phosphatase (TRAP) staining showed that both CaAl‐LDH and CM/LDH markedly reduced the number and area of TRAP‐positive multinucleated osteoclasts, whereas pure Ce‐MOF exerted limited inhibitory effects (Figure 2a,b). Consistent with this observation, cytoskeletal staining revealed well‐developed actin‐ring structures in the control (Ctrl) and Ce‐MOF groups, while CaAl‐LDH and CM/LDH treatment disrupted osteoclast spreading and cytoskeletal organization (Figure 2c). Subsequently, bovine bone slices were utilized to evaluate the effect of the materials on osteoclast‑mediated bone resorption. Abundant resorption pits were observed on the bone‑slice surfaces in the Ctrl and Ce‑MOF groups; the resorption area and depth were markedly higher than those of the CaAl‑LDH and CM/LDH groups (Figure S10). These results suggest that the LDH‐containing systems, especially CM/LDH, suppress osteoclast differentiation, maturation and function, which is likely associated with the acid‐buffering capacity of the CaAl‐LDH component.

The formation of a sealing zone is essential for osteoclast‐mediated bone resorption because it supports the localized acidification and enzyme accumulation required for bone matrix degradation [42]. To further evaluate whether CM/LDH affects osteoclast resorptive function, F‐actin and vinculin were co‐stained to visualize podosome belt organization. In the control group, osteoclasts exhibited large cell areas and continuous podosome belts with evident colocalization between F‐actin and vinculin. In contrast, CM/LDH‐treated osteoclasts showed reduced cell size, disrupted podosome belt structures, and weakened overlap between F‐actin and vinculin signals (Figure 2d). Line‐scan analysis further confirmed the decreased fluorescence colocalization of these two cytoskeletal components after CM/LDH treatment (Figure 2e). These findings indicate that CM/LDH impairs osteoclast cytoskeletal maturation and sealing‐zone integrity, thereby potentially limiting osteoclast resorptive activity.

Since osteoclast differentiation and function are closely associated with intracellular and pericellular acidification, the effect of CM/LDH on osteoclast pH regulation and survival was further investigated. Annexin V‐FITC/PI flow cytometry showed that CM/LDH treatment increased the proportion of apoptotic osteoclasts compared with the control group (Figure 2f,g). To explore whether this effect was related to acid neutralization, BCECF‐AM staining was used to monitor intracellular pH changes. CaAl‐LDH and CM/LDH significantly enhanced BCECF fluorescence intensity, indicating an increase in intracellular pH, whereas Ce‐MOF showed a weaker effect (Figure 2h,i). These data suggest that the CaAl‐LDH component can neutralize the acidic osteoclast‐associated microenvironment, thereby interfering with osteoclast maintenance and promoting osteoclast apoptosis. We also utilized acidic medium to investigate the effect of an acidic microenvironment on osteoclastogenesis. The results demonstrated that acidic medium promoted the differentiation and maturation of osteoclasts, whereas the addition of CM/LDH suppressed this effect. Nevertheless, substantial mature osteoclasts were still generated upon acidic medium replenishment in the CM/LDH‑treated group. Collectively, these findings indicate that CM/LDH attenuates osteoclast formation under pathological acidic microenvironments, and this protective effect is primarily attributed to its acid‑neutralizing capacity (Figure S11).

Collectively, CM/LDH regulates osteoclast fate through an acid‐neutralizing mechanism derived from its CaAl‐LDH “mother carrier” component. This regulation is reflected by reduced osteoclast differentiation, disrupted cytoskeletal and sealing‐zone organization, increased intracellular pH, and enhanced osteoclast apoptosis (Figure 2j). These results support the role of CM/LDH in attenuating excessive osteoclast activity and provide a cellular basis for its subsequent application in restoring bone‐remodeling balance under diabetic conditions.

### 2.3 CM/LDH Attenuates BMSCs Senescence and Restores Osteogenesis–Osteoclastogenesis Balance Under Diabetic Conditions

Excessive ROS and local acidification are key microenvironmental abnormalities that contribute to BMSC senescence, impaired osteogenic differentiation, and enhanced osteoclastogenesis in diabetic bone defects. Having established that CM/LDH integrates ROS‐scavenging and acid‐buffering activities, we next examined whether this mother‐child nanoplatform could alleviate diabetic microenvironment‐induced cellular dysfunction and restore osteogenesis–osteoclastogenesis balance in vitro.

**FIGURE 3:**
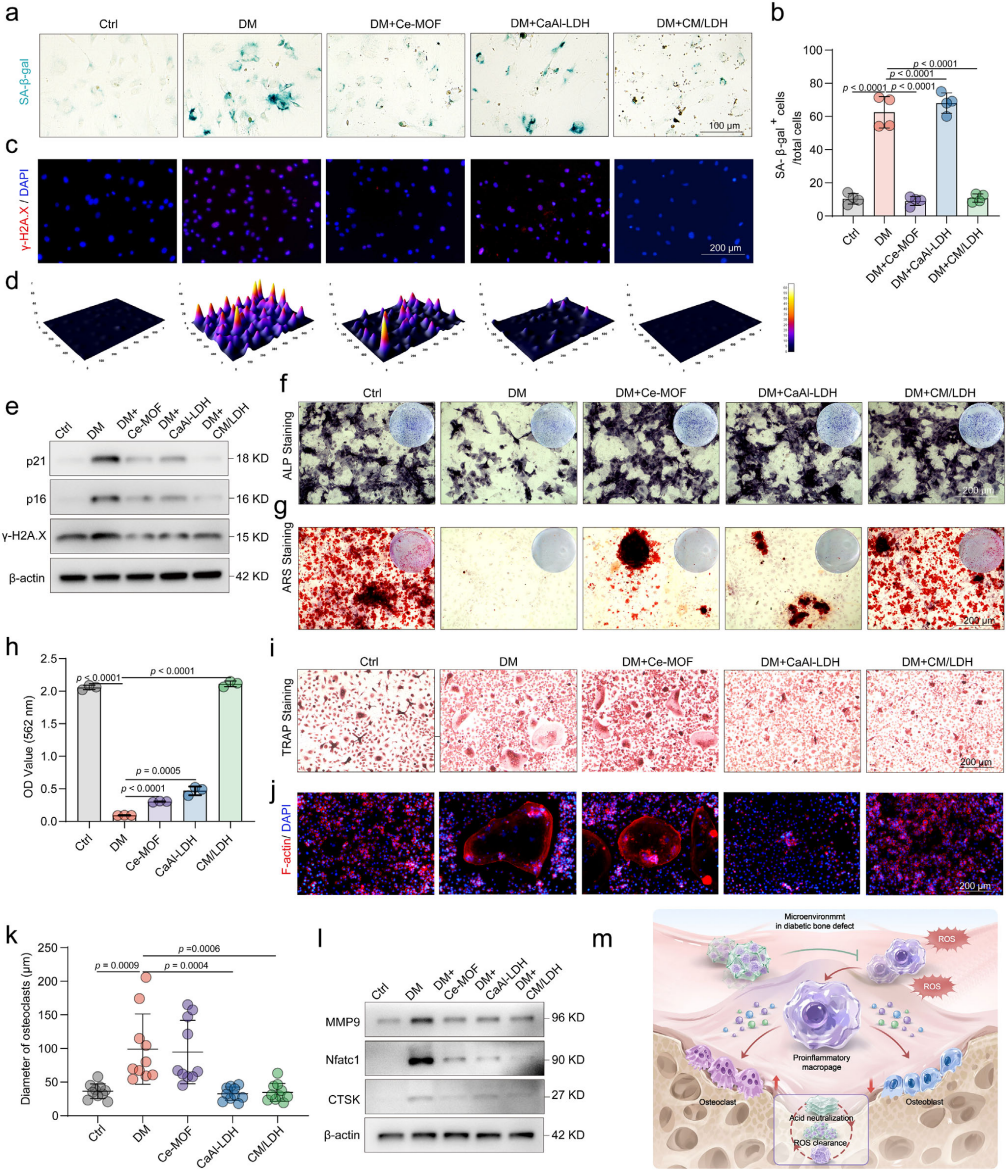
CM/LDH restores osteogenesis–osteoclastogenesis balance under diabetic microenvironment in vitro. (a,b) Representative SA‐β‐gal staining images and quantitative analysis in BMSCs of different groups. (c,d) Representative γ‐H2A.X immunofluorescence staining images and 3D surface plots of fluorescence intensity in BMSCs of different groups. (e) Western blotting of the senescence‐related markers under different interventions. (f) Representative images of ALP staining of BMSCs under different interventions. (g,h) Representative ARS staining images and quantitative analysis of mineralized matrix deposition in BMSCs after different treatments. (i) Representative TRAP staining images of osteoclast under different interventions. (j) Representative cytoskeletal fluorescence images of osteoclast under different interventions. (k) Quantitative analysis of osteoclast area based on TRAP staining. (l) Western blot analysis of osteoclast‐related markers under different interventions. (m) Schematic diagram of CM/LDH regulating osteogenesis‐osteoclastogenesis balance under diabetic microenvironment. Data in bar plots are presented as the mean ± SD. n ≥ 3 biologically independent samples. Statistical significance was determined by one‐way ANOVA.

The intracellular antioxidant effect of CM/LDH was first evaluated using DCFH‐DA fluorescent staining under H_2_O_2_‐induced oxidative stress. Compared with the H_2_O_2_‐treated group, Ce‐MOF and CM/LDH markedly reduced intracellular green fluorescence, whereas CaAl‐LDH showed a weaker effect, indicating that the Ce‐MOF‐containing systems effectively alleviated intracellular ROS accumulation (Figure S9c,d). Furthermore, we compared the cellular ROS‑scavenging performance between CM/LDH and the physical mixture of Ce‑MOF and CaAl‑LDH (Ce‐MOF + CaAl‐LDH). As shown in Figure S12, the physically‑mixed group exhibited substantially inferior capacity in both total intracellular ROS elimination and specific mitochondrial ROS scavenging relative to the CM/LDH group. These results reveal that the outstanding intracellular ROS‑quenching capability of CM/LDH originates not only from the intrinsic catalytic function of embedded Ce‑MOF, but also benefits from the electronic coupling effect between CaAl‑LDH and Ce‑MOF. Since excessive ROS is closely associated with cellular senescence and DNA damage, the anti‐senescent effect of CM/LDH was further examined in BMSCs under high‐glucose conditions. Senescence‐associated β‐galactosidase (SA‐β‐Gal) staining revealed that both Ce‐MOF and CM/LDH alleviated senescence in BMSCs exposed to high‐glucose pathological conditions (Figure 3a,b). Consistently, γ‐H2A.X immunofluorescence staining showed enhanced DNA damage signals under diabetic conditions, which were attenuated after Ce‐MOF and CM/LDH treatment (Figure 3c,d). Western blot (WB) analysis further confirmed that the expression levels of p21, p16, and γ‐H2AX were increased in the diabetic (DM) group and decreased after CM/LDH treatment (Figure 3e). These results suggest that CM/LDH alleviates high‐glucose‐induced BMSC senescence and DNA damage, which is closely associated with its ROS‐scavenging capability.

Because BMSC senescence directly compromises osteogenic potential, the effect of CM/LDH on BMSC proliferation and osteogenic differentiation was subsequently investigated. Under normal culture conditions, CaAl‐LDH and CM/LDH promoted ALP activity and mineralized nodule formation, supporting the osteogenesis‐favorable role of Ca^2^^+^ release from LDH‐containing materials (Figure S13). Under high‐glucose conditions, BMSC proliferation and osteogenic differentiation were markedly impaired, as reflected by reduced EdU‐positive cells, weakened ALP staining, and decreased ARS staining in the diabetic group (Figure S14 and Figure 3f–h). Treatment with Ce‐MOF or CaAl‐LDH alone only partially improved these defects, whereas CM/LDH more effectively restored BMSC proliferation, ALP activity, and mineralized matrix deposition. These findings indicate that CM/LDH rescues BMSC osteogenic function under diabetic conditions by combining Ce‐MOF‐mediated oxidative stress reduction with CaAl‐LDH‐mediated osteogenic support.

In addition to impairing osteogenesis, the diabetic microenvironment also enhances osteoclastogenesis and further disrupts bone‐remodeling balance [43]. Because osteoclasts originate from the monocyte–macrophage lineage, BMDMs isolated from diabetic and control mice were used for in vitro osteoclastic induction to evaluate diabetes‐associated osteoclastogenesis and its response to CM/LDH treatment. TRAP staining showed that BMDMs from diabetic mice generated more and larger osteoclasts than those from control mice, indicating enhanced osteoclastogenic potential under diabetic conditions (Figure 3i,k). Cytoskeletal fluorescence staining further revealed more developed osteoclast morphology and actin organization in the diabetic group (Figure 3j). Ce‐MOF treatment showed limited inhibition of diabetic osteoclastogenesis, whereas CaAl‐LDH‐containing treatments reduced osteoclast formation, with CM/LDH showing a more pronounced inhibitory effect (Figure 3i–k). Western blot analysis of osteoclast‐related proteins further supported that CM/LDH attenuated the elevated osteoclastic activity associated with the diabetic microenvironment (Figure 3l). Together, these results demonstrate that CM/LDH helps restore osteogenesis–osteoclastogenesis balance under diabetic conditions through the coordinated action of its two functional components. The Ce‐MOF “child drone” reduces oxidative stress‐associated BMSC senescence and supports osteogenic recovery, while the CaAl‐LDH “mother carrier” contributes to osteogenic support and osteoclast suppression through Ca^2^^+^ release and acid‐neutralizing activity. This coordinated regulation provides an in vitro cellular basis for the use of CM/LDH in diabetic bone defect repair (Figure 3m).

### 2.4 CM/LDH Mitigates Pathological Crosstalk Among BMDMs, BMSCs, and Osteoclasts Under Diabetic Conditions

**FIGURE 4:**
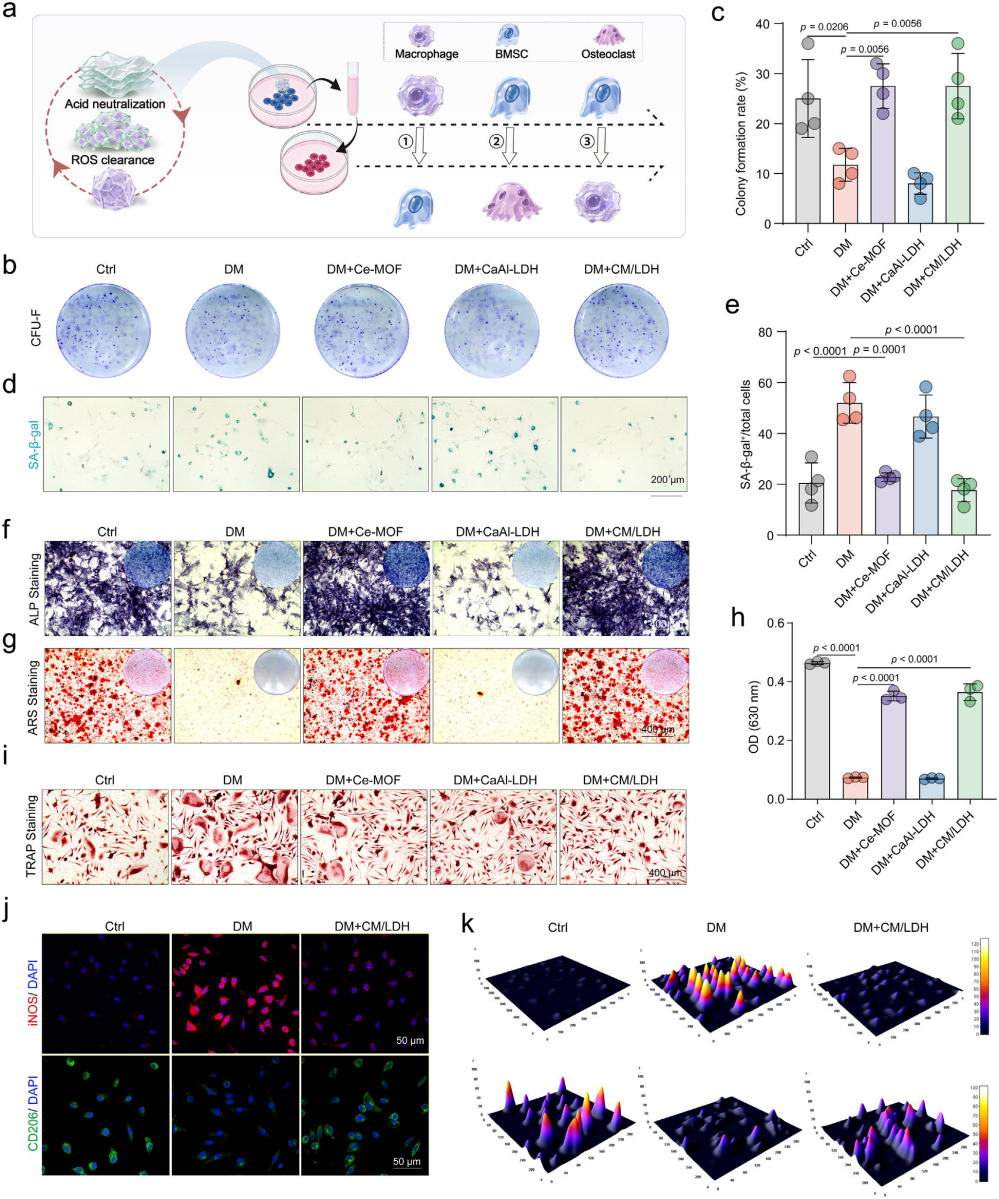
CM/LDH attenuates pathological intercellular crosstalk under diabetic microenvironment in vitro. (a) Schematic diagram of indirect co‐culture system used to evaluate intercellular crosstalk among BMDMs, BMSCs, and osteoclast‐lineage cells. (b,c) Representative images of cell colonies and quantitative analysis of BMSCs under different interventions. (d,e) Representative SA‐β‐gal staining images and quantitative analysis of BMSCs under different interventions. (f) Representative images of ALP staining of BMSCs under different interventions. (g,h) Representative ARS staining images and quantitative analysis of BMSCs after different treatments. (i) Representative TRAP staining images of osteoclasts under different interventions. (j,k) Representative iNOS and CD206 immunofluorescence staining images and 3D surface plots of fluorescence intensity in BMDMs after different treatments. Data in bar plots are presented as the mean ± SD. n ≥ 3 biologically independent samples. Statistical significance was determined by one‐way ANOVA.

Bone defect repair depends on coordinated communication among immune cells, BMSCs, and osteoclast‐lineage cells [44]. Under diabetic conditions, however, excessive ROS, inflammatory activation, and osteoclastogenic signals may disturb this intercellular communication and amplify bone‐remodeling imbalance. Having shown that CM/LDH protects BMSCs and suppresses osteoclastogenesis under diabetic conditions, we next established an indirect co‐culture system to evaluate whether CM/LDH could mitigate pathological crosstalk among BMDMs, BMSCs, and osteoclasts (Figure 4a).

To examine macrophage‐to‐BMSC communication, BMDMs isolated from diabetic and control mice were treated with different materials, and their culture supernatants were collected as conditioned media for BMSC culture. As shown in Figure 4b,c, conditioned media derived from diabetic BMDMs markedly reduced the number of BMSC colonies, indicating that diabetic BMDMs released soluble factors that impaired BMSC proliferation. Compared with the DM group, conditioned media from Ce‐MOF‐ and CM/LDH‐treated BMDMs improved BMSC colony formation, suggesting that attenuation of ROS‐associated macrophage dysfunction could reduce the inhibitory effect of diabetic BMDMs on BMSCs. SA‐β‐Gal staining further demonstrated that the CM/LDH nanoplatform alleviated the proportion of senescent BMSCs induced by diabetic BMDM‐conditioned medium (Figure 4d,e). Consistently, cytoskeleton staining, ALP staining, and ARS staining confirmed that CM/LDH‐treated BMDM‐conditioned medium better preserved BMSC morphology and osteogenic activity under diabetic conditions (Figure S15 and Figure 4f–h). These results indicate that CM/LDH alleviates the pathological signals transmitted from diabetic BMDMs to BMSCs and thereby supports BMSC proliferation and osteogenic function.

The reverse regulatory axis from BMSCs to BMDMs was then examined. Culture supernatants from BMSCs under different treatments were collected and applied to BMDMs during osteoclastic induction. TRAP staining revealed that BMDMs incubated with conditioned media from diabetic BMSCs exhibited enhanced fusion and osteoclastic differentiation compared with the control group (Figure 4i). This phenomenon was likely attributed to excessive ROS accumulation in the diabetic microenvironment, which induced extensive BMSC senescence [45]. Diabetic BMSCs may acquire a pro‐osteoclastogenic secretory phenotype, likely related to oxidative stress‐induced senescence and inflammatory signaling [27]. Compared with the DM group, conditioned media from Ce‐MOF‐ or CaAl‐LDH‐treated BMSCs reduced osteoclast formation, while CM/LDH produced a more evident inhibitory effect (Figure 4i). This effect may arise from the combined actions of Ce‐MOF‐mediated ROS scavenging, which alleviates BMSC senescence‐associated secretory cues, and CaAl‐LDH‐mediated acid buffering, which weakens the acidic conditions favorable for osteoclastogenesis.

In addition to osteoclastogenesis, the effect of BMSC‐derived conditioned media on BMDM polarization was further evaluated. Immunofluorescence staining for iNOS and CD206 indicated that CM/LDH successfully inhibited the pro‐inflammatory polarization of BMDMs driven by diabetic BMSCs (Figure 4j,k). These results suggest that CM/LDH not only reduces the pro‐osteoclastogenic influence of diabetic BMSCs, but also mitigates BMSC‐driven inflammatory polarization of BMDMs.

Collectively, these results demonstrate that CM/LDH exerts synergistic effects of ROS scavenging and acid neutralization to remodel the pathological crosstalk among macrophages, BMSCs and osteoclasts, and re‐establish normal intercellular communication between functional cells.

### 2.5 CM/LDH Suppresses Macrophage Inflammation Through Maintaining Mitochondrial Homeostasis

The indirect co‐culture experiments suggested that soluble paracrine factors released by diabetic BMDMs can transmit inflammatory stress to BMSCs, thereby contributing to BMSC dysfunction and osteogenesis–osteoclastogenesis imbalance. To further clarify how CM/LDH modulates macrophage inflammatory activity, we next focused on mitochondrial homeostasis, a key regulator of innate immune activation and inflammatory cytokine production in macrophages [46].

**FIGURE 5:**
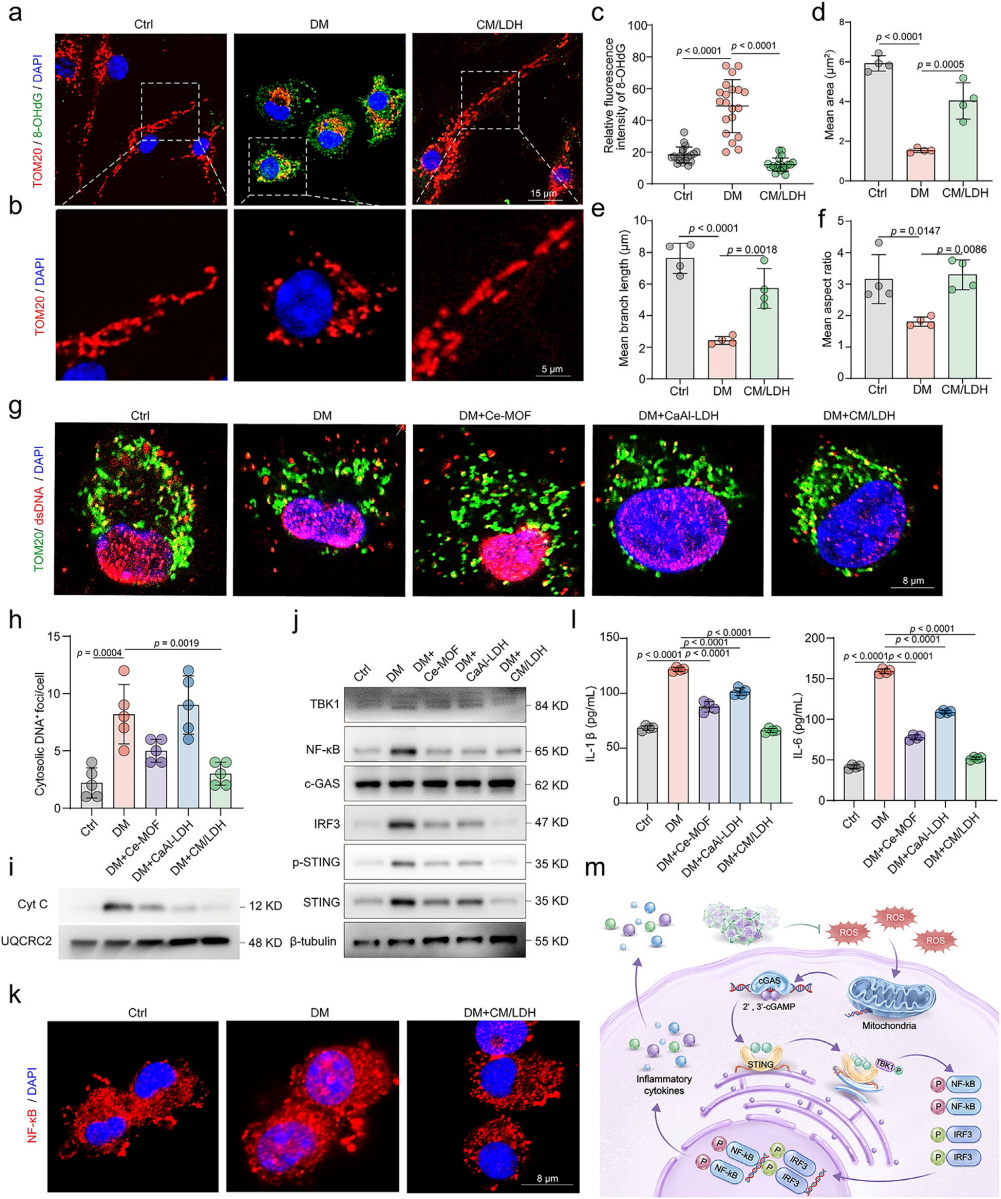
CM/LDH suppresses macrophage inflammation by maintaining mitochondrial homeostasis. (a) Representative immunofluorescence images of TOM20 and 8‐OHdG in BMDMs after different treatments. (b) Magnified images showing mitochondrial morphology in BMDMs after different treatments. (c) Quantitative analysis of 8‐OHdG immunofluorescence intensity in BMDMs. (d–f,) Quantitative analysis of mitochondrial morphology, including mean mitochondrial area, branch length, and aspect ratio. (g) Representative immunofluorescence images of BMDMs co‐stained with anti‐TOM20 (green), dsDNA (red), and DAPI (blue). (h) Quantitative analysis of cytosolic mtDNA in BMDMs under different conditions. (i) Representative western blot images showing Cyt C levels in the cytosolic fraction of BMDMs. UQCRC2 was included as a mitochondrial marker for evaluation of subcellular fractionation. (j) Western blot analysis of cGAS‐STING pathway‐related proteins in BMDM under different interventions. (k) Representative immunofluorescence images of NF‐κB in BMDMs under different conditions. (l) ELISA analysis of IL‐1β and IL‐6 levels in BMDM culture supernatants after different treatments. (m) Schematic illustration of CM/LDH‐mediated regulation of the mitochondrial homeostasis–mtDNA–cGAS‐STING inflammatory axis in macrophages. Data in bar plots are presented as the mean ± SD. n ≥ 3 biologically independent samples. Statistical significance was determined by one‐way ANOVA.

Mitochondrial oxidative injury in BMDMs was first evaluated by immunofluorescence staining of TOM20 and 8‐OHdG. TOM20 is a mitochondrial outer membrane translocase and is widely used as a marker to visualize mitochondrial localization and network morphology, whereas 8‐OHdG is a representative oxidative DNA damage marker generated by ROS‐mediated guanine oxidation. Therefore, increased 8‐OHdG signals colocalized with TOM20‐positive mitochondria indicate enhanced mitochondrial oxidative damage. Compared with BMDMs from the control group, diabetic BMDMs showed increased 8‐OHdG fluorescence colocalized with TOM20‐positive mitochondria, indicating enhanced mitochondrial oxidative damage under diabetic conditions (Figure 5a,c). Mitochondrial morphology analysis further showed evident fragmentation in diabetic BMDMs, accompanied by decreases in mean mitochondrial area, branch length, and aspect ratio (Figure 5b,d–f). After CM/LDH treatment, mitochondrial oxidative damage signals were attenuated, and mitochondrial morphology partially shifted toward an elongated and interconnected state in diabetic BMDMs. The MitoSOX fluorescence staining images of BMDMs in each group also demonstrated that compared with the DM group and CaAl‑LDH‑treated groups, CM/LDH and Ce‑MOF alleviated excessive mitochondrial oxidative stress (Figure S16). These results suggest that CM/LDH alleviates diabetic microenvironment‐associated mitochondrial oxidative injury and helps preserve mitochondrial homeostasis in macrophages.

Mitochondrial homeostasis disruption can promote the leakage of mitochondrial DNA (mtDNA) into the cytosol, thereby triggering innate immune inflammatory signaling [47]. To examine whether CM/LDH affects this process, TOM20 and dsDNA immunofluorescence staining was performed to visualize mitochondria‐associated DNA distribution. In diabetic BMDMs, dsDNA signals were separated from TOM20‐positive mitochondria and accumulated in the cytosolic region, indicating increased mtDNA leakage (Figure 5g,h). Ce‐MOF and CaAl‐LDH treatment showed partial effects, whereas CM/LDH more effectively reduced cytosolic mtDNA signals. In addition, three‐dimensional reconstructed fluorescence images obtained using an mtDNA‐targeting fluorescent probe further showed that diabetic conditions induced abnormal cytosolic redistribution of mitochondrial DNA in BMDMs, whereas this mtDNA leakage/redistribution was attenuated by CM/LDH treatment (Figure S17). Mitochondrial membrane potential, measured via JC‑1 staining, showed evident depolarization in BMDMs from the DM and CaAl‑LDH groups. In contrast, Ce‑MOF‑ and CM/LDH‑treated BMDMs retained membrane potential comparable to control levels, reflecting intact mitochondrial function (Figure S18). Western blot analysis of cytosolic cytochrome c (Cyt C) further showed that Cyt C was markedly released into the cytoplasm in BMDMs from the diabetic group, indicating impaired mitochondrial membrane integrity (Figure 5i). In contrast, only CM/LDH treatment effectively attenuated this change, suggesting that CM/LDH could inhibit cytosolic leakage of Cyt C and help maintain mitochondrial functional stability.

Since cytosolic mtDNA is an important trigger of the cGAS‐STING pathway, the activation of this inflammatory signaling axis was further examined [22, 23, 48]. WB analysis showed that high‐glucose stimulation did not markedly alter cGAS expression in BMDMs, but significantly upregulated the expression and activation of its downstream proteins including STING, TBK1, IRF3 and NF‐κB (Figure 5j). CM/LDH treatment reduced the levels of these downstream pathway proteins to the baseline of the control group, indicating that CM/LDH effectively suppressed diabetes‐associated cGAS‐STING pathway activation in BMDMs. Consistently, NF‐κB immunofluorescence staining showed enhanced nuclear translocation of NF‐κB in diabetic BMDMs, whereas CM/LDH treatment attenuated this translocation pattern (Figure 5k). The inflammatory output downstream of this pathway was then evaluated by Enzyme‐linked immunosorbent assay (ELISA). Compared with the control group, diabetic BMDMs secreted higher levels of IL‐1β and IL‐6, while CM/LDH treatment significantly decreased the secretion of these proinflammatory cytokines (Figure 5l).

Collectively, CM/LDH regulates macrophage inflammatory responses through a mitochondrial homeostasis‐centered mechanism. By reducing ROS‐associated mitochondrial oxidative injury, CM/LDH limits cytosolic mtDNA leakage and subsequently attenuates activation of the cGAS‐STING inflammatory pathway, thereby decreasing proinflammatory cytokine secretion from diabetic BMDMs (Figure 5m).

### 2.6 CM/LDH Promotes Mandibular Defect Repair Under Diabetic Conditions

To determine whether the microenvironment‐regulatory effects of CM/LDH could be translated into clinically relevant craniofacial bone repair, mandibular defect models were established in diabetic db/db mice and non‐diabetic db/m mice. The db/db model was selected because it exhibits persistent obesity‐associated hyperglycemia and impaired bone healing, thereby providing a stringent platform for assessing diabetic bone regeneration [49]. The mandible was selected because mandibular and alveolar bone defects are highly prevalent in dental extraction, periodontal disease, implant failure, trauma, tumor resection, and reconstructive surgery, and their repair is particularly compromised by diabetes‐associated oxidative stress, inflammation, infection susceptibility, and abnormal bone remodeling [50]. Moreover, GelMA photocrosslinkable hydrogel was selected as the carrier for loading CM/LDH and applied to bone defect sites owing to its favorable properties including biocompatibility and shape adaptability. As shown in Figure S19, the storage modulus (G′) of the GelMA + CM/LDH group was consistently higher than the loss modulus (G′′), indicating that CM/LDH did not interfere with the formation of a stable three‑dimensional elastic network by GelMA. Moreover, the incorporation of CM/LDH slightly increased the storage modulus of the GelMA hydrogel, demonstrating that CM/LDH could act as a filler to serve as physical cross‑linking junctions and improve the mechanical performance of the hydrogel. Furthermore, the DCFH‑DA staining assay also verified that the GelMA hydrogel as a carrier did not compromise the antioxidant function of CM/LDH (Figure S20).

**FIGURE 6:**
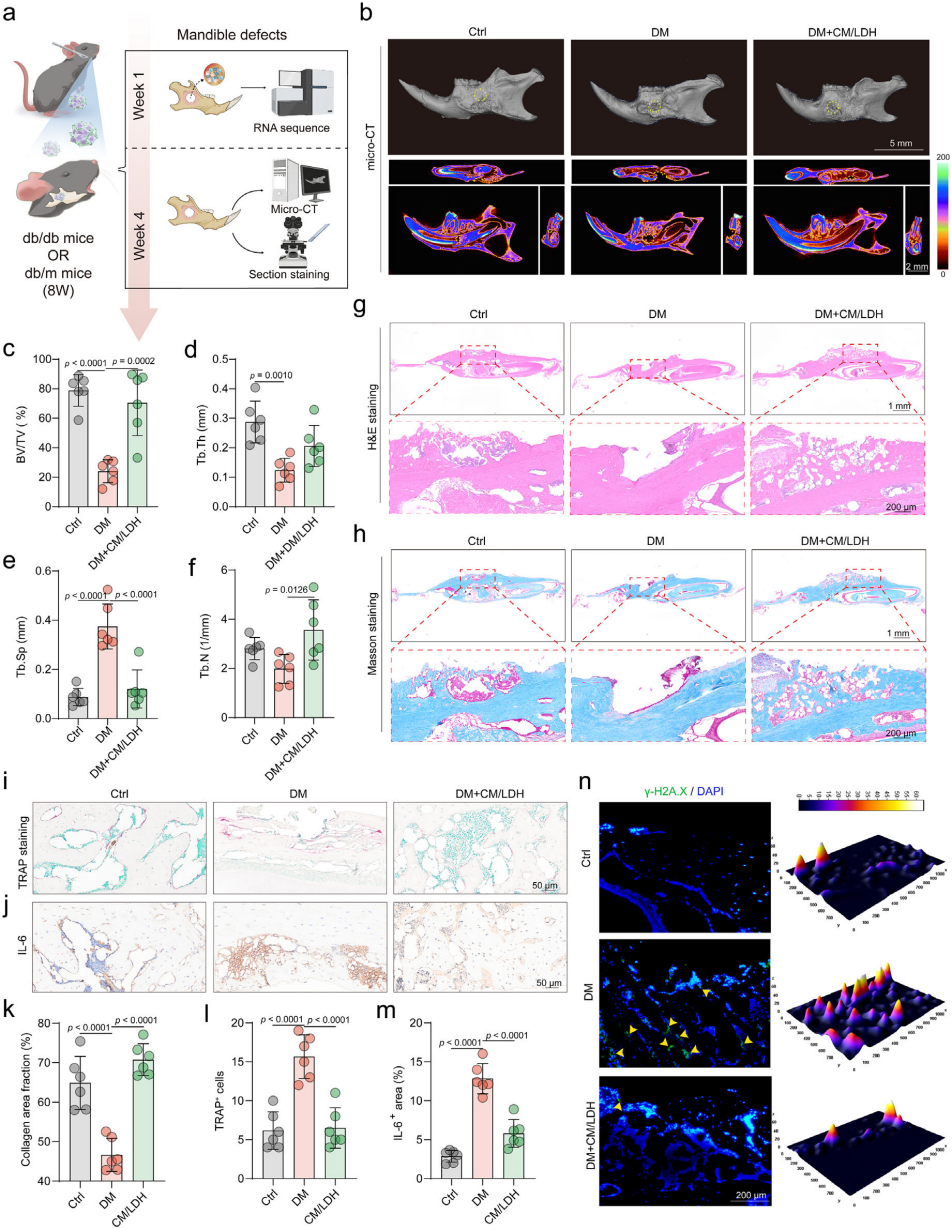
CM/LDH promotes mandibular defect repair in diabetic mice. (a) Schematic diagram illustrating mandibular bone defect model in db/db mice or db/m mice and the corresponding treatment procedure. (b) Representative micro‐CT images of the mandibular defect sites in different groups at 4 weeks after treatments. The yellow dashed area indicates the bone defect region. (c–f) Quantitative analysis of BV/TV, Tb.Th, Tb.Sp and Tb.N in mandibular defect regions at 4 weeks after treatment (n = 6). (g) Representative H&E staining images of the newly formed bone within the mandibular defects. (h) Representative Masson's trichrome staining images of the mandibular defect regions. (i) Representative TRAP staining images of mandibular defect regions. (j) Representative immunohistochemical staining of IL‐6 in mandibular bone defect regions. (k) Quantitative analysis of collagen area in mandibular defect regions at 4 weeks after treatment. (l) Quantitative analysis of TRAP‐positive cells of mandibular sections. (m) Quantitative analysis of immunohistochemical staining of IL‐6 in mandibular bone defect regions. (n) Representative immunofluorescence staining images of γ‐H2A.X and 3D thermal image analysis in mandibular defects. Data in bar plots are presented as the mean ± SD. n ≥ 6 biologically independent samples. Statistical significance was determined by two‐tailed unpaired t‐test or by one‐way ANOVA.

Local treatments were applied to the defect sites, and bone regeneration and pathological microenvironmental changes were evaluated at different time points (Figure 6a). Micro‐computed tomography (micro‐CT) analysis at 4 weeks after surgery revealed that the untreated diabetic defects remained clearly visible and were not completely bridged, indicating limited spontaneous repair within the 4‐week observation period. In contrast, more newly formed bone was observed within the defect region after CM/LDH treatment (Figure 6b). Quantitative analysis further showed that CM/LDH increased bone volume fraction (BV/TV) and trabecular thickness (Tb.Th) to 2.8‐ and 1.8‐fold of those in the DM group, respectively (Figure 6c,d). Meanwhile, trabecular separation (Tb.Sp) decreased by 62%, while the trabecular number (Tb.N) rose to 1.9‐fold compared with the untreated DM group (Figure 6e,f). These results indicate that local CM/LDH administration improves bone regeneration and trabecular microarchitecture in diabetic mandibular defects.

Histological staining was then performed to further evaluate tissue repair within the defect region. H&E staining showed evident residual defect areas and limited tissue filling in the diabetic group, whereas the CM/LDH‐treated defects exhibited more continuous newly formed bone‐like tissue (Figure 6g). Masson's trichrome staining further indicated that greater collagen deposition in the CM/LDH group compared with the DM group, suggesting improved matrix formation and maturation during defect repair (Figure 6h,k). TRAP staining showed that CM/LDH effectively reduced the number of TRAP‐positive cells in the bone defect area relative to the DM group (Figure 6i,l). Immunohistochemical staining for IL‐6 showed stronger inflammatory signals in the diabetic group, which were attenuated after CM/LDH treatment (Figure 6j,m). Given that oxidative stress status and angiogenesis at diabetic bone‑defect sites are critical determinants for bone regeneration, it is necessary to assess how the material modulates these key factors within the defect microenvironment [51]. Immunohistochemical analyses of 8‐OHdG and CD31 demonstrated that CM/LDH alleviated excessive oxidative stress at diabetic bone defect sites and promoted angiogenesis upon amelioration of the local microenvironment (Figures S21 and S22). In addition, γ‐H2A.X immunofluorescence staining revealed increased DNA damage‐associated signals in diabetic defect tissues, while CM/LDH treatment reduced γ‐H2A.X‐positive signals in the defect region (Figure 6n). These results suggest that CM/LDH not only directly promotes bone formation, but also accelerates bone repair by indirectly ameliorating multiple aspects of the osteogenic microenvironment.

The preliminary biosafety of CM/LDH was further evaluated by histological analysis of major organs after local implantation in mandibular defects of wild‐type C57 mice. H&E staining of the heart, liver, spleen, lung, and kidney showed no obvious histological abnormalities at 4 weeks after CM/LDH administration (Figure S23). Together, these in vivo results indicate that local application of CM/LDH can alleviate cellular senescence, mitigate inflammation, restore the balance between osteogenesis and osteoclastogenesis, and ultimately accelerate the healing of diabetic bone defects.

### 2.7 Transcriptomic Analysis Reveals CM/LDH‐Mediated Remodeling of the Diabetic Bone Defect Microenvironment

**FIGURE 7:**
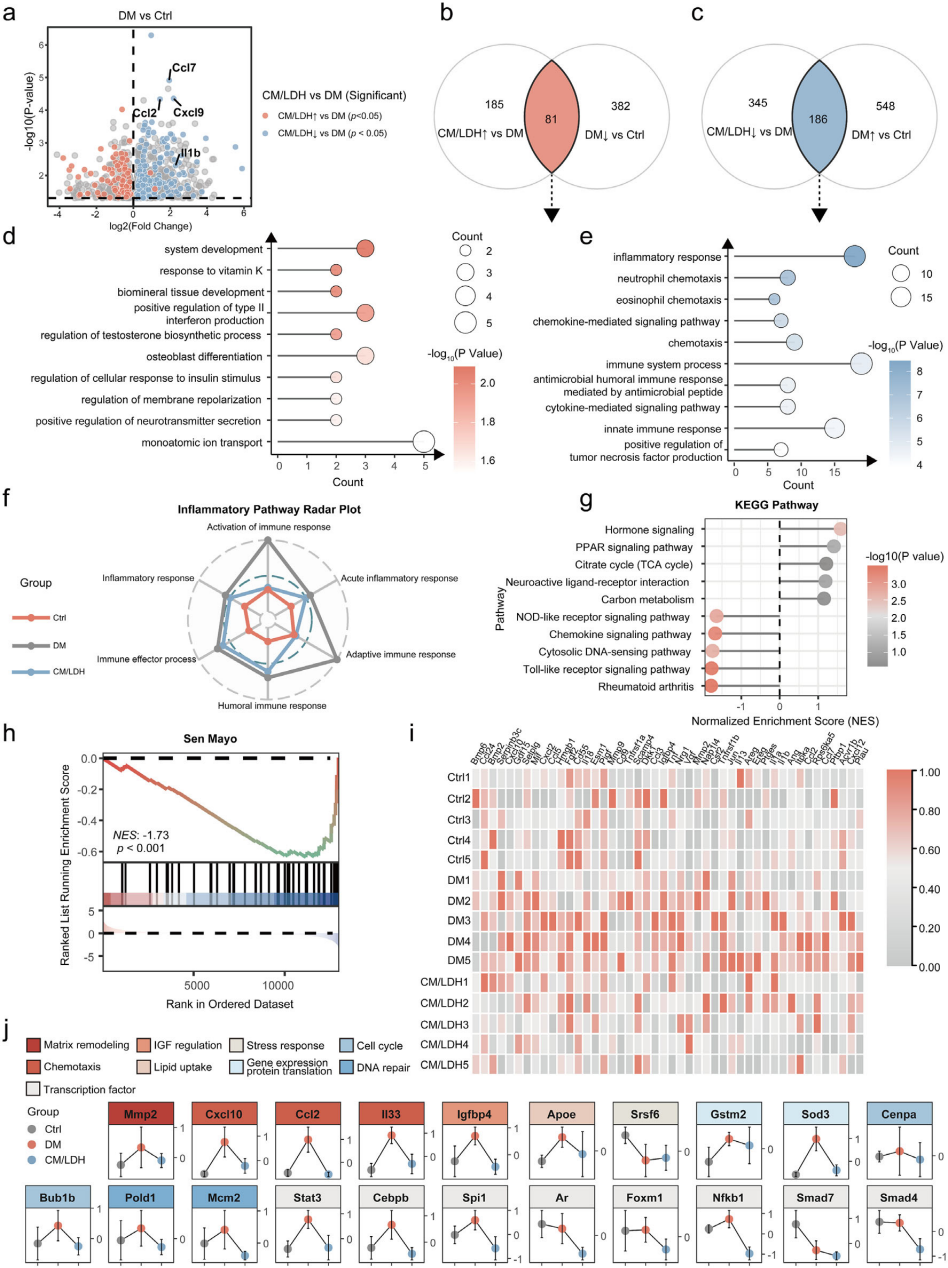
Mechanistic Exploration of CM/LDH‐Regulated In Vivo Bone Regeneration. (a) Volcano plot of gene expression in DM vs Ctrl. Genes significantly altered in CM/LDH vs DM (*p* < 0.05, DESeq2) are highlighted: red, upregulated; blue, downregulated. (b, c) Venn diagrams showing overlaps of significantly altered genes: (b) genes upregulated in CM/LDH vs DM and downregulated in DM vs Ctrl; (c) genes downregulated in CM/LDH vs DM and upregulated in DM vs Ctrl. (d,e) GO Biological Process enrichment analysis of the overlapping genes from (b) and (c), respectively. (f) The ssGSEA scores of inflammatory biological processes were min‐max scaled to facilitate cross‐group comparison. (g) KEGG‐based GSEA showing pathway‐level changes in the CM/LDH group compared with the DM group. (h) GSEA enrichment plot for the SenMayo senescence‐associated gene signature. (i) Heatmap of gene expression levels for genes in the SenMayo signature across groups. (j) Line plots showing expression profiles of key SASP‐related genes across the three groups. Data in bar plots are presented as the mean ± SD. n ≥ 3 biologically independent samples. Statistical significance was determined by one‐way ANOVA analysis.

To further explore the molecular basis underlying CM/LDH‐mediated bone regeneration in vivo, transcriptomic sequencing was performed on mandibular defect tissues at the early repair stage after model establishment. As shown in Figure S24, distinct gene expression profiles were observed between diabetic db/db mice and non‐diabetic db/m mice, indicating that the diabetic microenvironment substantially altered the transcriptional landscape of bone defect tissues. After local CM/LDH implantation into the bone defects of db/db mice, a subset of diabetes‐associated differentially expressed genes showed partial reversal toward the control pattern (Figure 7a). Representative inflammatory genes, including Ccl7, Ccl2, Cxcl9, and IL‐1β, were markedly downregulated in the CM/LDH group, suggesting attenuation of inflammatory activation in diabetic bone defects.

To further identify biological processes associated with CM/LDH‐mediated repair, genes that were downregulated in the DM group relative to the Ctrl group but upregulated after CM/LDH treatment were analyzed. Gene Ontology (GO) enrichment analysis showed that these genes were mainly associated with biomineral tissue development and osteoblast differentiation (Figure 7b,d). Conversely, genes that were upregulated in the DM group but downregulated after CM/LDH treatment were enriched in activation of inflammatory responses, immune cell chemotaxis, chemokine‐mediated signaling pathway and cytokine‐mediated signaling pathway (Figure 7c,e). Consistently, inflammatory pathway scoring further confirmed that CM/LDH broadly reduced multiple immune and inflammatory response signatures that were enhanced in diabetic defect tissues (Figure 7f). These results indicate that CM/LDH shifts the transcriptional profile of diabetic bone defects from an inflammatory state toward a more repair‐associated state.

Kyoto Encyclopedia of Genes and Genomes (KEGG) pathway analysis was then performed to further evaluate pathway‐level changes. Compared with the DM group, CM/LDH treatment was associated with increased enrichment of metabolism‐related pathways, including the PPAR signaling pathway and the tricarboxylic acid cycle. (Figure 7g). In contrast, several inflammatory and innate immune pathways were reduced after CM/LDH treatment, including the NOD‐like receptor signaling pathway, chemokine signaling pathway, cytosolic DNA‐sensing pathway, and Toll‐like receptor signaling pathway. These pathway changes are consistent with the previous cellular findings that CM/LDH alleviates mitochondrial stress, limits mtDNA‐associated inflammatory signaling, and suppresses macrophage inflammatory activation under diabetic conditions. We also performed gene‑set enrichment analysis (GSEA) on tissue mitochondrial‑related pathways. The results revealed that the CM/LDH group exhibited significantly enhanced enrichment in mitochondrial respiratory chain complex I assembly, ATP synthesis, and inner mitochondrial membrane‑associated signatures compared with the DM group, demonstrating that the material could effectively restore mitochondrial homeostasis in tissues at the bone‑defect sites (Figure S25).

The SenMayo gene set, a robust biomarker for senescence monitoring with promising clinical potential, enables high‐fidelity identification of senescent cells across different tissues and species, characterizes senescence signatures, and depicts core intercellular signaling pathways [52]. Gene set enrichment analysis (GSEA) results indicated that CM/LDH remarkably suppressed the expression of the SenMayo gene set in bone defect tissues of db/db mice (Figure 7h). Heatmap of representative signature genes further confirmed that CM/LDH restored the transcriptional patterns of the DM group to levels comparable to the Ctrl group (Figure 7i). The expression profiles of representative SASP‐related genes were further compared among the three groups. Compared with the DM group, CM/LDH treatment reduced the expression of several SASP‐related genes, although the changes varied among individual genes (Figure 7j). Together, these bulk transcriptomic results suggest that CM/LDH treatment reshapes the diabetic bone defect microenvironment toward a repair‐favorable state, as reflected by reduced inflammatory signatures, enhanced repair‐associated biological processes, and decreased senescence‐associated transcriptional features. Importantly, these findings should be interpreted as tissue‐level evidence consistent with, rather than direct in vivo validation of, the macrophage‐specific mechanism identified in vitro.

Despite these encouraging findings, several limitations should be considered. First, the present study focused on a 4‐week repair period, and the long‐term degradation, tissue retention, systemic distribution, ion‐release behavior, and chronic biosafety of CM/LDH remain unclear. Second, although the mandible is directly exposed to the oral environment, an oral infection or periodontitis‐associated diabetic defect model was not included. Third, the in vivo mechanism was mainly inferred from histology and bulk transcriptomics; therefore, cell‐type‐specific mechanistic studies are needed to verify the roles of macrophages, BMSCs, osteoblasts, and osteoclasts in CM/LDH‐mediated repair. Fourth, although acidic PBS is a simplified system and cannot fully reproduce the complex biochemical composition of diabetic bone defects, including proteins, lactate, glucose, inflammatory mediators, and cellular metabolism, it provides a controllable model for comparing the intrinsic acid‐buffering capacity of different materials. Future studies combining in situ pH imaging and spatiotemporal monitoring of local acidosis will be necessary to precisely define the dynamic pH changes during diabetic bone repair. In addition, the dynamic matching between CM/LDH‐mediated acid buffering/antioxidant functions and the pathological timeline of diabetic bone defects still requires further validation through time‐course analysis of local pH, ROS, inflammation, and material degradation in vivo. Finally, the current in vivo validation was limited to a mouse model, and large‐animal studies are necessary to further evaluate translational potential.

## 3 Conclusion

In conclusion, this work constructed a multifunctional mother‐child nanoplatform (CM/LDH) to overcome the obstacle of impaired craniofacial bone regeneration under diabetic pathological microenvironment. Benefiting from the intrinsic acid‐neutralizing property of the CaAl‐LDH mothership and the osteoinductive capability of released Ca^2^^+^, CM/LDH remodels the acidic niche of diabetic bone defects and synchronously balances osteogenic and osteoclastic metabolism. As loaded drone units, Ce‐MOF is protected against acidic inactivation by the mothership, thereby exerting effective ROS‐scavenging activity within diabetic bone defects, regulate bone immunity, and interrupt pathological cellular crosstalk. In vitro results demonstrated that CM/LDH inhibits osteoclastic activity, alleviates BMSCs senescence, enhances their osteogenic potential and modulates macrophage inflammatory responses under diabetic conditions. Further mechanistic studies revealed that CM/LDH preserves mitochondrial homeostasis and reduces mtDNA leakage in macrophages, which restrains the activation of the cGAS‐STING inflammatory pathway and subsequently suppresses the production and secretion of pro‐inflammatory cytokines. In vivo assays using diabetic mouse mandibular defect models confirmed that this nanoplatform alleviates local inflammation, restricts osteoclast differentiation, facilitates cellular rejuvenation and bone formation, and remarkably accelerates diabetic bone repair. The potential advantage of CM/LDH lies in its integrated functional design, in which acid buffering by CaAl‐LDH and ROS scavenging by Ce‐MOF may cooperatively improve the diabetic bone defect microenvironment and subsequent cellular responses. Accordingly, this platform represents a promising candidate for the treatment of diabetic craniofacial bone defects, particularly in mandibular defects where systemic diabetic dysregulation intersects with the unique oral inflammatory, microbial, and mechanical microenvironment. More broadly, the present work highlights a disease‐microenvironment‐oriented design concept that may inspire the development of multifunctional biomaterials for other pathological bone defects involving oxidative stress, chronic inflammation, local acidification, or osteoimmune imbalance. However, the direct translation of CM/LDH to other bone defects requires further validation.

## 4 Experimental Section

Experimental Section/Methods are available in the Supporting Information.

## Author Contributions

**Xu Chen**: conceptualization, investigation, Writing – original draft, methodology, validation, visualization, Writing – review and editing, software, formal analysis. **Xingyu Zhu**: writing – original draft, investigation, methodology, validation, visualization, writing – review and editing, software. **Zhixin Qiu**: validation, visualization, formal analysis. **Yijia Zhu**: methodology, visualization, writing – review and editing. **Xinhui Zheng**: methodology, investigation, formal analysis. **Dongqi Fan**: methodology, formal analysis, visualization. **Jinglei Zhan**: methodology, visualization, software. **Congxiu Mao**: validation, writing – review and editing, software. **Ping Ji**: conceptualization, project administration, supervision, data curation. **Tao Chen**: conceptualization, funding acquisition, supervision, resources. **Qingqing He**: conceptualization, funding acquisition, project administration, supervision.

## Funding

This work was financially supported by National Outstanding Youth Science Fund Project of National Natural Science Foundation of China (32322044), Joint Funds of the National Natural Science Foundation of China (U25A2099), Special Funding Program for Postdoctoral Research Projects of Chongqing (2025CQBSHTB2018), China Postdoctoral Science Foundation (2024M763903), Graduate Scientific Research Innovation Project, College of Stomatology, Chongqing Medical University (KQY202513, KQY202402), Chongqing Municipal Graduate Scientific Research Innovation Project (CYB25230, CYS240318).

## Conflicts of Interest

The authors declare no conflicts of interest.

## Supporting information




**Supporting File**: advs77841‐sup‐0001‐SuppMat.docx.

## Data Availability

The data that support the findings of this study are available from the corresponding author upon reasonable request.
