# Supplementary material for: Hierarchical Nanoplatform Integrating Oxidative–Acidic Microenvironment Remodeling and Bone Homeostasis Modulation for Diabetic Bone Defect Therapy
Source: Adv Sci (Weinh). 2026 Sep 27:e77841. Online ahead of print. doi: 10.1002/advs.77841 (PMC13616312; doi:10.1002/advs.77841)
Supplement: Supplementary file 1 [file ADVS-9999-e77841-s001.docx]

Supporting Information

Hierarchical Nanoplatform Integrating Oxidative–Acidic Microenvironment Remodeling and Bone Homeostasis Modulation for Diabetic Bone Defect Therapy

Xu Chen, Xingyu Zhu, Zhixin Qiu, Yijia Zhu, Xinhui Zheng, Dongqi Fan, Jinglei Zhan, Congxiu Mao, Ping Ji*, Tao Chen*, Qingqing He*

X. Chen, X. Zhu, Z. Qiu, Y. Zhu, X. Zheng, D. Fan, J. Zhan, C. Mao, T. Chen, Q. He

The Affiliated Stomatological Hospital of Chongqing Medical University, Chongqing Key Laboratory of Oral Diseases, Chongqing Municipal Key Laboratory of Oral Biomedical Engineering of Higher Education, Chongqing Municipal Health Commission Key Laboratory of Oral Biomedical Engineering, Chongqing, China

E-mail: chentao1985@hospital.cqmu.edu.cn; heqingqing@hospital.cqmu.edu.cn

P. Ji

The Affiliated Stomatological Hospital of Chongqing Medical University, The Affiliated Yongchuan Hospital of Chongqing Medical University, Chongqing Key Laboratory of Oral Diseases, Chongqing Municipal Key Laboratory of Oral Biomedical Engineering of Higher Education, Chongqing Municipal Health Commission Key Laboratory of Oral Biomedical Engineering, Chongqing, China

E-mail: jiping@hospital.cqmu.edu.cn

**Materials and methods**

**Preparation of CaAl-LDH, Ce-MOF and CM/LDH**

To synthesize CaAl-LDH, calcium nitrate (Sigma, Cat. No. C1396, USA) and aluminum nitrate (Macklin, Cat. No. 7784-27-2, China) were added into 100 mL of ethanol/water solution (1:1) at a molar ratio of 2:1. Subsequently, 0.2 M sodium hydroxide (prepared in 50% aqueous ethanol, Sigma, Cat. No. 221465, USA) was added to adjust the pH of the system to 10 under nitrogen protection, 70 °C oil bath, and magnetic stirring at 1000 rpm. After 24 hours of reaction, the mixture was centrifuged at 11000 rpm for 10 minutes to collect the product, which was then washed with deionized water three times. The obtained precipitate was freeze-dried to yield CaAl-LDH.

Ce-MOF was synthesized according to a previously reported method [1]. Under magnetic stirring, 0.177 g of terephthalic acid (Aladdin, Cat. No. P108506, China) was dissolved completely in 24 mL of N,N-dimethylformamide (General-reagent®, Cat. No. G76259B, China). Subsequently, 8 mL of aqueous solution containing 0.558 g of ammonium cerium nitrate (Aladdin, Cat. No. A305310, China) was added. After stirring for 10 minutes, the resulting mixture was transferred into a Teflon-lined autoclave and sealed. After naturally cooling to room temperature, the precipitate was collected by centrifugation and washing, and finally freeze-dried using a lyophilizer (SHANGHAI TIANFENG INDUSTRIAL CO., LTD, Model TF-FD-27S) to obtain Ce-MOF.

For the synthesis of CM/LDH, 0.5 g of lyophilized Ce-MOF and 0.1 g of CaAl-LDH were weighed and added into 30 mL of ethanol/water solution (1:1). Then, 0.2 M sodium hydroxide (prepared in 50% aqueous ethanol) was added to adjust the pH of the system to 10. The mixture was magnetically stirred at room temperature for 24 h. The precipitate was collected by centrifugation and washing, and finally freeze‑dried using a lyophilizer to obtain CM/LDH.

**Characterization**

The surface morphology and elemental distribution of the nanomaterials were characterized using a SU8010 field emission scanning electron microscope (FE-SEM) equipped with an energy dispersive X-ray spectroscopy (EDX) detector (Hitachi, Japan). The zeta potential and hydrodynamic size of each nanoparticle was measured with a Zetasizer Nano ZS90 analyzer (Malvern Instruments, UK). The surface chemical composition and ionic valence states of the samples were determined by an ESCALAB 250Xi X-ray photoelectron spectrometer (XPS, Thermo Fisher Scientific, USA). The chemical bonds and functional group characteristics of the materials were analyzed using a Nicolet iS50 Fourier transform infrared spectrometer (FT‑IR, Thermo Fisher Scientific, USA). The crystal structure of the nanoparticles was investigated using an X'Pert Powder X-ray diffractometer (PANalytical, Netherlands). The thermal stability and component composition of the materials were characterized using a thermogravimetric analyzer (HITACHI STA200, Japan). The elemental contents were determined by an inductively coupled plasma‑optical emission spectrometer (ICP‑OES, Avio 550 Max, PerkinElmer, USA). Cyclic voltammetry (CV) and electrochemical impedance spectroscopy (EIS) were measured using an electrochemical workstation (Zahner Zennium XC, German). A three‑electrode system was utilized, where the sample‑modified glassy carbon electrode served as the working electrode, a Pt sheet as the counter electrode, and an Ag/AgCl electrode as the reference electrode in the corresponding electrolyte. CV measurements were performed at an appropriate scan rate within a predefined potential window. EIS tests were carried out over the frequency range of 10⁻² Hz to 10⁵ Hz with an AC perturbation amplitude of 5 mV.

**CAT-like catalytic activity test**

A H₂O₂ assay kit (Solarbio, Cat. No. BC3590, China) was used in the experiment. The detailed procedure was as follows: microcentrifuge tubes were divided into groups (three replicates per group), followed by the sequential addition of 500 μL of 1 mM H₂O₂, 100 μL of each material solution, and 400 μL of ddH₂O. After mixing, the reaction was carried out in the dark for 30 min. After the reaction, the mixture was centrifuged at 12,000 rpm for 10 min, and the supernatant was collected for later use. Reagents and the prepared supernatant (or standard solution) were added sequentially according to the kit instructions, mixed well, and incubated in the dark for another 30 min. The precipitate was collected by centrifugation, dissolved in Reagent IV from the kit, and allowed to stand at room temperature for 5 min to obtain the test solution. The color of the solution was recorded, and the absorbance was measured at full wavelength and at 415 nm using a microplate reader.

To evaluate the oxygen production capacity of the materials, 1 mL of the material solution at a concentration of 0.625 mg/mL was added to 4 mL of phosphate-buffered saline (PBS) at pH 7.4 and mixed uniformly to form a homogeneous reaction system. Subsequently, 500 μL of 10 M hydrogen peroxide (Sigma, Cat. No. 108600, USA) was added to initiate the oxygen production reaction. A dissolved oxygen meter (Leici, Model DZS-706F, China) was used to monitor the dissolved oxygen concentration in the reaction system in real time. The entire reaction lasted for 15 min, and dissolved oxygen data were recorded every minute.

**SOD-like catalytic activity test**

The SOD-like activity of the materials was evaluated using a WST-8-based superoxide dismutase assay. Briefly, different material suspensions were incubated with the WST-8 working solution and xanthine oxidase reaction system according to the manufacturer’s instructions (Beyotime, Cat. No. S0101M, China). In this assay, superoxide anions generated by the xanthine/xanthine oxidase system reduce WST-8 to a water-soluble formazan dye, which shows absorbance at 465 nm. Materials with SOD-like activity inhibit formazan formation by scavenging superoxide anions. After a reaction period of 20–30 min, the absorption spectra of the reaction supernatant were measured over the wavelength range of 230–900 nm using a microplate reader.

**Acid neutralization test**

A defined volume of phosphate-buffered saline (PBS, pH 4.5) was added into reaction vessels separately. Then, under magnetic stirring, 50 mg/mL CaAl-LDH, Ce-MOF, CM/LDH suspensions, or 0.1 M and 0.01 M NaOH solutions were added dropwise gradually at 10 μL per aliquot. After each addition, a pH meter (Leici, Model PHSJ-6L, China) was used to monitor the pH value of the reaction system continuously in real time, and the instantaneous pH value after each titration was recorded.

**Isolation and culture of bone marrow mesenchymal stem cells ( BMSCs)**

BMSCs were isolated according to a previously reported method [2]. Three-week-old C57BL/6 mice were sacrificed by cervical dislocation and disinfected by immersion in 75% (v/v) ethanol for 5 min. Femora and tibiae of bilateral hind limbs were dissected in a biosafety cabinet, and attached muscles and soft tissues were removed. After washing with PBS, the bones were transferred to complete medium. The epiphyses were cut off to expose the bone marrow cavity, and the bone marrow was flushed repeatedly with medium until the bone tissue appeared pale white. The remaining bone was minced into 1–3 mm³ fragments and digested with 1 mg/mL collagenase II digestion solution (GIBCO, 17101015, USA) with shaking at 37 ℃ and 200 rpm for 1 h. After terminating digestion, the supernatant was discarded, and the bone chips were washed twice with α-MEM. The bone chips were then seeded into T25 culture flasks and cultured in α-MEM medium (Hyclone, SH30265.01B, USA) supplemented with 10% fetal bovine serum (FBS, Thermo Fisher, 12664025C, USA) and 1% penicillin-streptomycin (Solarbio, P1400, China). The flasks were incubated at 37 ℃ in a 5% CO₂ incubator for 3 days. After 3 days, the complete medium was replaced to remove non-adherent cells, and culture was continued until day 5, with medium changes or passaging performed according to cell confluence. For passaging, pre-warmed trypsin (GIBCO, 25300054, USA) was added for digestion for 40 s. The flask bottom was tapped gently to suspend cells, complete medium was added to terminate digestion, and cells were centrifuged, resuspended, and subcultured at a ratio of 1:2. Third-generation BMSCs were uniformly used for subsequent experiments. For cell culture of the control group, BMSCs were seeded as required and cultured in normal-glucose α-MEM medium containing 5.5 mM D-glucose for 5 days. For in vitro cell culture of the diabetic group and material group, cells were incubated in high-glucose α-MEM medium supplemented with 25 mM D-glucose for 5 days after seeding, with or without the experimental material (250 μg mL^-1^) for co-culture. Upon completion of treatment, the culture supernatants were collected or relevant indicators were detected following standard experimental protocols.

**Isolation and culture of bone marrow derived macrophages（BMDMs）**

BMDMs were isolated from tibiae and femora harvested from 15-week-old diabetic mice or age-matched wild-type C57BL/6 mice [3]. Under a biological safety cabinet, bone marrow cells were flushed out from the bone marrow cavity using sterile PBS, and the cell suspension was collected. The collected cell suspension was centrifuged at 1200 rpm and 4 ℃ for 4 min, and the supernatant was discarded after centrifugation. An appropriate amount of red blood cell lysis buffer (Solarbio, R1010, China) was added to the pellet, and the cells were gently resuspended by pipetting, followed by centrifugation at 500 rcf and 4 ℃ for 5 min. The supernatant was discarded, and the red blood cell lysis step was repeated once to sufficiently remove erythrocytes. PBS containing 2% penicillin–streptomycin was added to the treated cell pellet. After gentle resuspension, the mixture was centrifuged at 500 rcf and 4 ℃ for 3 min, and the supernatant was discarded to wash the cells. Corresponding complete medium containing macrophage colony‑stimulating factor (M‑CSF, SinoBiological, 51112‑MNAH, China) was added to the cell pellets of different groups. For the control group, the cell culture medium consisted of RPMI 1640 basal medium (Gibco, 12633020, USA) containing 5mM glucose, supplemented with 10% FBS and 1% penicillin‑streptomycin. For the DM group, the medium was prepared with the same components except that glucose was additionally added to a final concentration of 25mM. The final concentration of M‑CSF was prepared by adding 0.8 μL of 100 μg/mL M‑CSF stock solution per milliliter of medium. The cells were gently resuspended and evenly dispersed, then cultured in an incubator at 37 ℃ with 5% CO₂. On day 2 of culture, the supernatant of each flask was gently aspirated and transferred to new T25 flasks for continued culture. On day 3, 2 mL of complete medium was added to each flask, and M‑CSF was supplemented according to the current total volume of medium in each flask. Cells in each group were further cultured until day 5, then seeded into 24‑well plates at a density of 2×10⁵ cells per well. Cells were cultured until day 7 with or without materials, and the culture supernatants were collected for subsequent conditioned-medium experiments.

**Osteoclast induction**

BMDMs isolated from Diabetic and control mice were seeded into 24‑well plates at a density of 2×10⁵ cells per well and cultured in their respective media (high glucose and normal glucose) for 24 h. The medium was then replaced with corresponding medium containing 50 ng/mL receptor activator of nuclear factor‑κB ligand (RANKL, SinoBiological, 50343‑M01H, China) and 25 ng/mL macrophage colony‑stimulating factor (M‑CSF, SinoBiological, 51112‑MNAH, China), with or without the addition of materials (250 μg mL^-1^) according to experimental requirements. The medium was changed every 2–3 days, and after continuous culture for 5 days, assays related to osteoclast activity were performed. In the indirect co-culture system, the collected BMSCs-conditioned media from each group were mixed with BMDM osteoclast differentiation medium at a ratio of 1:2 to treat BMDMs. The medium was refreshed every 2 days. After 5 days of osteoclast induction, samples were collected for osteoclast activity detection.

**Osteogenic induction of BMSCs**

BMSCs were seeded into 12‑well plates at a density of 5×10⁴ cells per well. After 24 h, the medium was replaced with osteogenic induction medium containing 50 ng/mL ascorbic acid (Solarbio, China), 10 mM β‑glycerophosphate (Solarbio, China), and 10 nM dexamethasone (Solarbio, China). In the indirect co‑culture system, culture supernatants of BMDMs from each group were mixed with BMSC osteogenic induction medium at a ratio of 1:2and used to culture BMSCs [4]. The culture medium for each group was changed every 2–3 days, and samples were collected at corresponding time points for osteogenic activity assays.

**Cell Viability Assay**

Cell viability was measured using Cell Counting Kit‑8 (CCK‑8) on days 1, 3, and 7, respectively. BMSCs were seeded into 96‑well plates at a density of 5000 cells per well and treated with nanomaterials at various concentrations. After incubation for 1, 3, and 7 days, cells were washed with phosphate‑buffered saline (PBS), followed by the addition of medium containing 10% CCK‑8 reagent (Beyotime Biotechnology, China). Cells were further incubated at 37 °C for 2 hours. Finally, absorbance at 450 nm was measured using a microplate reader (PerkinElmer, USA), and viability was calculated accordingly.

Cell viability was assessed using a Calcein/Propidium Iodide (PI) Viability/Cytotoxicity Assay Kit (Beyotime Biotechnology, Shanghai, China). BMSCs were seeded into 24‑well plates at a density of 2×10⁴ cells per well. After 24 h of culture, cells were treated with 250 μg mL^-1^ of the material for 72 h. The culture medium was discarded, and the staining working solution was prepared according to the manufacturer’s instructions. Then, 0.25 mL of the staining working solution was added to each well.Following incubation at 37 ℃ for 30 min, cells were observed using an EVOS FL Auto Cell Imaging System (Thermo Fisher, USA).

**Assays for Alkaline Phosphatase (ALP) Activity and Osteogenic Mineralization**

ALP activity was detected in bone marrow‑derived mesenchymal stem cells (BMSCs) after 7 days of culture/co‑culture. The culture medium in the plate was aspirated, and cells were washed twice with PBS. 4% paraformaldehyde (PFA) was added to fix the cells for 10 min, followed by two additional washes with PBS. Solution A was prepared as follows: 1 mL of 1 M Tris buffer (pH 9.0), 250 μL of 2 M MgCl₂·H₂O, and 200 μL of 5 M NaCl were combined and adjusted to a final volume of 10 mL with ddH₂O. Solution A was applied to the cells and incubated for 15 min. Solution B was freshly prepared in the dark: 20 μL of NBT (Sigma, Germany) and 15 μL of BCIP (Sigma, Germany) were added to 4 mL of Solution A. Solution B was then added to the cells and incubated at 37 ℃ for 30 min in the dark. Finally, cells were washed twice with ddH₂O and observed using an EVOS FL Auto Cell Imaging System.

After 3 weeks of culture/co‑culture, alizarin red S (ARS) staining solution (Solarbio, China) was used to evaluate the mineralization degree of cultured cells. The culture medium in the plate was aspirated, and cells were washed twice with PBS. PFA was added to fix the cells for 10 min, followed by two additional washes with PBS. ARS staining solution was added and incubated at room temperature for 60 min. The staining solution was discarded, cells were washed twice with ddH₂O, and images were captured using an EVOS FL Auto Cell Imaging System. For quantitative analysis, alizarin red‑stained deposits were dissolved in 10% (w/v) cetylpyridinium chloride solution (Sigma, Germany). After incubation, absorbance at 620 nm was measured using a microplate reader (PerkinElmer, USA).

**Colony formation assay**

To evaluate the colony‑forming ability of mouse BMSCs, the following assay was performed. Mouse BMSCs in the logarithmic growth phase were digested with trypsin and prepared into a single‑cell suspension. After adjusting the cell concentration, cells were seeded into 6‑well plates at a density of 1000 cells per well, cultured in complete medium containing conditioned medium, and incubated in a cell culture incubator at 37 °C with 5% CO₂ and saturated humidity for 10 days. The medium was changed every 3 days during the culture period. At the end of the culture, the medium was discarded, and cells were gently washed twice with phosphate‑buffered saline (PBS). Cells were fixed with 4% paraformaldehyde fixative solution at room temperature for 30 min. After fixation, the fixative was removed, cells were washed twice with PBS, and stained with 1% crystal violet staining solution (Solarbio, Cat. No. G1062) at room temperature for 20 min. The plates were then gently rinsed with distilled water until the background was colorless and air‑dried naturally. Colonies were counted using ImageJ software (National Institutes of Health, USA). Only colonies containing ≥50 cells were counted. Each sample was set up in triplicate, the experiment was independently repeated three times, and the average number of colonies was calculated.

**Intracellular ROS measurement**

RAW264.7 cells were seeded into 24‑well plates at a density of 1×10⁵ cells per well. For the H₂O₂ and material groups, 700 μM H₂O₂ was added to stimulate the cells for 12 h. Intracellular reactive oxygen species (ROS) levels were then detected using the DCFH‑DA fluorescent probe (Beyotime, Cat. No. S0033S) following the manufacturer’s instructions. Briefly, RAW264.7 cells were incubated with DCFH‑DA working solution (diluted 1:1000) at 37 ℃ for 20 min in the dark. After washing twice with serum‑free medium, intracellular ROS content was measured using an EVOS FL Auto Cell Imaging System (Thermo Fisher, USA) at an excitation/emission wavelength of 488/525 nm.

**Detection of mitochondrial ROS**

Following various treatments, cells were incubated with 5 μM MitoSOX Red mitochondrial superoxide indicator (MeilunBio, MB12813, China) at 37 °C for 20 min in the dark. After washing with pre‑warmed PBS, cell nuclei were counter‑stained with Hoechst. Confocal laser‑scanning microscopy was used to capture fluorescence images.

**JC‑1 staining for mitochondrial membrane potential measurement**

Following various treatments, BMDM cells were incubated with JC‑1 working solution (MeilunBio, MA0338, China) at 37 °C for 20 min in the dark. The working solution was prepared according to the manufacturer’s instructions. After removal of the staining solution, cells were washed twice with pre‑warmed PBS. Fluorescent images were captured by laser scanning confocal microscopy.

**Cytoskeleton staining**

Cytoskeleton (microfilaments) was examined by phalloidin fluorescence staining (Beyotime, Cat. No. C2203S, China) according to the manufacturer’s protocol. The culture medium of osteogenically induced BMSCs or osteoclastogenically induced BMDMs was discarded, and the cells were washed three times with pre‑cooled PBS (pH 7.4) for 5 min each. Then the cells were fixed with 4% paraformaldehyde at room temperature in the dark for 15 min, followed by three washes with PBS. Subsequently, the cells were incubated with immunostaining permeabilization solution (Triton X‑100, Beyotime Biotechnology, China) at room temperature for 30 min. After three washes with PBS, the cells were blocked with 5% goat serum for immunostaining (Meilun Bio, China) for 30 min. Actin‑Tracker Red staining solution diluted at 1:100 was added directly, and the cells were incubated at 37 ℃ in the dark for 1 h. Unbound probes were removed by three washes with PBS. An appropriate amount of DAPI staining solution (Beyotime, Cat. No. C1005, China) was added, and the cells were incubated at room temperature in the dark for 15 min, followed by three final washes with PBS. After staining, cells were observed and photographed under an EVOS FL Auto Cell Imaging System.

**Enzyme-linked immunosorbent assay (ELISA)**

Culture supernatants of BMDMs after different treatments were collected, and the concentrations of interleukin‑6 (IL‑6) and interleukin‑1β (IL‑1β) were measured using a Mouse IL‑6 ELISA Kit (BYabscience, Cat. No. BY‑EM220188, China) and a Mouse IL‑1β ELISA Kit (BYabscience, Cat. No. BY‑EM220174, China), respectively, following the manufacturer’s instructions. A double‑antibody sandwich enzyme‑linked immunosorbent assay (ELISA) was employed. Calibration standards and test samples were added to microplate wells pre‑coated with anti‑IL‑1β and anti‑IL‑6 capture antibodies (solid‑phase antibodies). Subsequently, HRP‑conjugated anti‑mouse IL‑1β antibody and HRP‑conjugated anti‑mouse IL‑6 antibody (enzyme‑labeled detection antibodies) were added. After incubation and thorough washing to remove unbound components, a sandwich complex of solid‑phase antibody–antigen–enzyme‑labeled antibody formed on the solid surface of the microplate. Substrate solutions A and B were then added. Under the catalysis of HRP, the substrates produced a blue chromogenic product, which was finally converted to yellow upon the addition of stop solution (acidic solution). The optical density (OD value) was measured at 450 nm using a microplate reader. The concentrations of IL‑1β and IL‑6 in the samples were calculated by fitting the calibration curve.

**Detection of osteoclast apoptosis**

Osteoclast apoptosis was detected using an Annexin V‑FITC/PI Apoptosis Detection Kit (Meilun Bio, Cat. No. MA0220, China). Osteoclasts obtained after induction were treated under different conditions for 24 hours, respectively. After collecting single cells, the cells were washed by gentle shaking or pipetting with pre‑cooled PBS, and harvested by centrifugation for a total of two washes. 1× Binding buffer working solution was added to the cell pellet to resuspend the cells at a density of 1×10⁶ cells/mL. 100 μL of the cell suspension (containing 1×10⁵ cells) was transferred to a new tube, followed by the addition of 5 μL Annexin V‑FITC and 10 μL PI. After gentle mixing, the cells were incubated at room temperature in the dark for 15 minutes. The apoptosis level of osteoclasts was analyzed using a flow cytometer (BD LSRFortessa, BD Biosciences, USA).

**Detection of intracellular pH in osteoclasts**

Intracellular pH changes in osteoclasts were detected using the BCECF AM pH fluorescent probe (Beyotime Biotechnology, Cat. No. S1006, China). Osteoclasts obtained after induction were treated under different conditions for 24 hours. After discarding the culture medium and washing twice with PBS, the cells were stained with BCECF AM working solution (5 μM) at 37 °C for 30 minutes. After washing with PBS, cells were observed and photographed under an EVOS FL Auto Cell Imaging System, and fluorescence intensity was measured at an excitation wavelength of 488 nm and an emission wavelength of 535 nm.

**Animals**

Wild-type (WT) C57BL/6J mice, aged 6 weeks, were purchased from the Laboratory Animal Center of Chongqing Medical University. Male db/m and db/db mice at 6 weeks of age were obtained from Chongqing Huasu Tongjie Laboratory Animal Sales Co., Ltd. All animals were routinely housed until 8 weeks of age prior to subsequent experiments. They were maintained in a specific pathogen-free (SPF) environment with a temperature of 24 °C, a 12 h/12 h light/dark cycle, and 50% humidity, with free access to food and water. All animal experimental procedures were performed in strict accordance with institutional guidelines and approved by the Ethics Committee of Stomatological Hospital of Chongqing Medical University (Animal Ethics Approval No.:CQHS-REC-2026 (LSNo.053)).

**Experimental animal models**

**Diabetic model building.** Six-week-old WT mice were adaptively fed for 1 week before being fed a high-fat diet (XTHF60, Jiangsu Xietong Pharmaceutical Bio-Engineering Co.,Ltd). Age-matched control mice were fed a standard chow diet for 4 consecutive weeks. Subsequently, mice in the HFD group received an intraperitoneal injection of streptozotocin (STZ, S8050, Solarbio, China) at a dose of 65 mg/kg for 5 consecutive days. STZ was dissolved in ice-cold citrate buffer (pH 4.5). Mice in the control group received an equal volume of citrate buffer as a sham treatment. Body weight, food intake, water intake, and urine output of mice were monitored daily during modeling. One week after the last injection, mice were fasted for 12 h, and fasting blood glucose (FBG) was measured. Diabetes was considered successfully established if FBG was > 11.1 mmol/L. Successfully established diabetic mice were utilized specifically for isolating BMDMs to investigate macrophage inflammatory activation, mitochondrial homeostasis, and cGAS-STING-related signaling under diabetic metabolic stress.

**Mandibular defect model.** The db/db mouse model was used as an independent in vivo diabetic bone defect model to evaluate mandibular bone regeneration under a stable hyperglycemic and obesity-associated diabetic background [5]. Age-matched db/m littermates were used as non-diabetic controls where applicable. Anesthesia was induced with 2.3% isoflurane gas, and maintained at 1%–2% via a nasal mask equipped with an exhaust gas recovery system during the operation. After removing local hair in the mandibular surgical area, an incision was made along the inferior border of the mandible to expose the buccal cortical bone. Using a round bur cooled with normal saline, two full‑thickness critical‑sized bone defects with a diameter of 1.2 mm were prepared on bilateral mandibles of each mouse. This defect size was selected based on previous mouse mandibular defect studies in which circular full-thickness mandibular defects of approximately 1.0–1.5 mm were commonly used to evaluate craniofacial bone regeneration [6]. The modeled mice were randomly divided into three groups with different treatments: Group 1 (control group): Mandibular defects of db/m mice were filled with gelatin methacryloyl (GelMA) photocurable hydrogel loaded with PBS; Group 2: Mandibular defects of db/db mice were filled with GelMa photocurable hydrogel loaded with PBS; Group 3: Mandibular defects of db/db mice were filled with GelMa photocurable hydrogel loaded with CM/LDH. Mice designated for tissue sequencing were euthanized 7 days postoperatively, and the implanted materials and surrounding tissues at the defect sites were harvested for sequencing analysis. For the remaining mice in each group, mandibular specimens were collected 4 weeks postoperatively for subsequent detection and analysis.

**Senescence-associated beta-galactosidase staining**

Senescence-associated β-galactosidase (SA-β-Gal) staining was performed using an SA-β-Gal staining kit (Beyotime Biotechnology, Shanghai, China) in strict accordance with the manufacturer’s instructions. In vivo experiments: Diabetic model mice were anesthetized and euthanized. Fresh spleen and thymus tissues were rapidly dissected and immediately fixed in 10% neutral buffered formalin for 3–4 hours. After fixation, tissues were transferred to 30% sucrose solution and dehydrated overnight at 4°C to balance tissue osmotic pressure. Dehydrated tissues were embedded in OCT compound (CellPath, UK), and 6–8 μm thick frozen sections were prepared using a cryostat and mounted on glass slides for subsequent staining. Prepared tissue sections were immersed in SA-β-Gal staining solution containing X-Gal and incubated overnight in a 37°C CO₂-free incubator for 16–24 hours to complete the staining reaction. In vitro cell experiments: Cells cultured to the logarithmic growth phase were gently washed twice with sufficient PBS to remove residual medium. The kit-provided fixative solution was added, and cells were incubated at room temperature for 5 minutes to achieve fixation. After fixation, cells were washed twice with PBS to remove fixative, followed by the addition of freshly prepared SA-β-Gal staining solution. Staining was completed by incubation under the same conditions as in vivo experiments (37°C, CO₂-free incubator) for 16–24 hours.

**Western blotting analysis**

Protein expression levels in cells and tissues were detected and analyzed by Western blot according to previously reported methods. The detailed procedures were as follows: Equal amounts of cell lysate or total tissue protein were separated by sodium dodecyl sulphate polyacrylamide gel electrophoresis (SDS-PAGE), and then electrically transferred onto 0.45 μm polyvinylidene fluoride membranes (PVDF membranes, Millipore, USA). The PVDF membranes were blocked in Tris-buffered saline containing Tween-20 (TBST, 100 mM Tris-HCl, pH 7.4) supplemented with 5% non-fat milk for 2 h at room temperature to block non-specific binding sites. After blocking, primary antibodies against p21 (HUABIO, HA500156, China), p16 (Proteintech, 60626-1, China), γ-H2A.X (HUABIO, ET1602-2, China), Cyt C (HUABIO, ET1610-60, China), MMP-9 (Proteintech, 30592-1-AP, China), Nfatc1 (Proteintech, 66963-1, China), CTSK (Proteintech, 11239-1-AP, China), TBK1 (HUABIO, HA601045, China), NF-κB (HUABIO, HA750058, China), cGAS (Proteintech, 29958-1-AP, China), IRF3 (HUABIO, ET1612-4, China), p-STING (Affinity Biosciences, AF7416, USA), STING (Cell Signaling, 13647, USA) and β‑actin (Proteintech, 66009-1, China) were added and incubated overnight at 4°C. On the next day, the PVDF membranes were washed three times with TBST buffer (5 min each) to remove unbound primary antibodies, followed by incubation with goat anti-rabbit or anti-mouse IgG secondary antibody (1:5000, ZSGB-Bio, Beijing, China) for 1.5 h at room temperature. Finally, protein band signals were detected using a gel imaging analysis system (Bio‑Rad, USA) combined with an ultrasensitive ECL chemiluminescence kit (BeyoECL Plus, Beyotime Biotechnology, Shanghai, China), and the staining intensity of each protein band was quantitatively analyzed using Image J software (NIH, Littleton, CO, USA).

**Cytoplasmic and mitochondrial protein extraction**

Cytoplasmic and mitochondrial proteins were extracted according to the instructions of the cytoplasmic and mitochondrial protein extraction kit. At least 1×10⁷ treated cells were collected and washed twice with pre‑cooled 1×PBS (pH 7.4), followed by centrifugation at 3000 rpm (800 g) for 5 min each time. One milliliter of cytoplasmic extraction buffer (supplemented freshly with 1 μL protease inhibitor, 5 μL phosphatase inhibitor, and 1 μL DTT per mL immediately before use) was added to the processed cell sample. The mixture was transferred to a glass homogenizer and homogenized 30–50 times on ice with continuous cooling. The homogenate was transferred to a pre‑cooled centrifuge tube, vigorously vortexed for 15 sec at maximum speed, and incubated on ice for 10–15 min. After centrifugation at 3000 rpm for 10 min at 4°C, the pellet was discarded. The supernatant was transferred to a new pre‑cooled centrifuge tube and centrifuged at 12000 rpm (12830 g) for 30 min at 4°C to pellet the mitochondrial fraction. The resulting supernatant, which was the cytoplasmic protein fraction, was transferred to a new tube and stored at −80°C for further use. To collect the mitochondrial pellet, 0.1 mL of freshly prepared cytoplasmic extraction buffer containing inhibitors was added, vortexed for 30 sec, and centrifuged at 13000 rpm (15000 g) for 10 min at 4°C. The supernatant was discarded. Then 0.1 mL of mitochondrial lysis buffer (supplemented freshly with 1 μL protease inhibitor, 5 μL phosphatase inhibitor, and 1 μL DTT per mL immediately before use) was added to the washed mitochondrial pellet. The mixture was incubated on ice for 30 min for lysis, vortexed again for 30 sec, and centrifuged at 13000 rpm (15000 g) for 10 min at 4°C. The supernatant was collected as the mitochondrial protein fraction.

**Targeted staining of mitochondrial DNA**

BMDMs were seeded at a density of 1×10⁵ cells per well in 24‑well plates containing cell slides. After various treatments, the cell culture medium was removed and cells were washed twice with PBS. The prepared Mito‑Tracker Deep Red FM working solution (1:5000 dilution, Beyotime Biotechnology, China) was added, followed by incubation at 37 °C for 20 min in the dark. After washing twice with PBS, Mito DNA‑targeting Tracker staining working solution (1:1000 dilution, Guangzhou Biolight Biotechnology Co., Ltd., Cat. No. MTD001, China) was added, and cells were incubated at 37 °C for 30 min in the dark. The staining solution was discarded, and cells were washed twice with pre‑warmed culture medium. Nuclear staining was performed using 5 μg/mL Hoechst 33342 solution (1:5000 dilution, Beyotime Biotechnology, China) at 37 °C for 15 min in the dark, followed by two washes with PBS. PBS was then added, and fluorescence imaging was captured using a Dragonfly 200 high‑speed spinning disk confocal imaging system (Andor, UK).

**Immunoﬂuorescence staining**

For cell samples, cells from each group were seeded onto cell slides and cultured until the detection time point after treatment under different conditions. The samples were washed twice with PBS and then fixed with 4% paraformaldehyde solution (Biosharp, China) at room temperature for 15 minutes. For tissue sections, deparaffinization was performed using xylene, followed by gradual rehydration with graded ethanol. Heat-induced antigen retrieval was conducted prior to blocking: sections were heated in antigen retrieval buffer (Beyotime Biotechnology, China) at 95 °C for 30 minutes to expose heat-mediated antigenic epitopes, whereas this step was omitted for cell samples. Subsequently, cell membrane permeabilization and immunoblocking were performed: cell samples were permeabilized with immunostaining permeabilization solution containing Triton X‑100 (Beyotime Biotechnology, China) for 30 minutes, followed by blocking with 5% normal goat serum (Meilun Bio, China) for 30 minutes; tissue sections were blocked with 5% normal goat serum at room temperature for 30 minutes after antigen retrieval.

Primary antibodies were prepared using Western primary antibody dilution buffer (Meilun Bio, China), added to the samples (cells and tissue sections), and incubated overnight at 4 °C. The primary antibodies used in this study were as follows: γ‑H2A.X (diluted 1:200, HUABIO, ET1602-2, China), TOM20 (diluted 1:200, HUABIO, ET1609-25, China), 8‑OHdG (diluted 1:500, Bioss, bs-1278R, China), NF-κB (diluted 1:200, HUABIO, HA750058, China), Vinculin (diluted 1:200, MCE, YA5517, USA), dsDNA (diluted 1:50, Santa Cruz, sc-58749, USA).

Then, fluorescently labeled secondary antibodies were added and incubated for 60 minutes at room temperature in the dark. The secondary antibodies used were as follows: Alexa Fluor 594-conjugated goat anti-rat IgG (diluted 1:200, Abcam, ab150160, USA), Alexa Fluor 488-conjugated goat anti-rabbit IgG (diluted 1:200, ZSGB-Bio, ZF-0511, China), Alexa Fluor 594-conjugated goat anti-mouse IgG (diluted 1:200, ZSGB-Bio, ZF-0513, China), Alexa Fluor 594-conjugated goat anti-rabbit IgG (diluted 1:200, ZSGB-Bio, ZF-0516, China), Alexa Fluor 488-conjugated goat anti-mouse IgG (diluted 1:200, ZSGB-Bio, ZF-0512, China). Nuclei were counterstained and mounted using antifade mounting medium with DAPI (Beyotime Biotechnology, China) at room temperature. Samples were washed three times with PBS between each incubation step. Microscopic observation and image acquisition were performed using a Dragonfly 200 high-speed spinning disk confocal imaging system (Andor, UK) or a VS200 automatic slide scanner (Olympus, Japan).

**Hematoxylin and Eosin (H&E) and Masson Staining**

After fixation with 4% paraformaldehyde, bone tissue specimens were decalcified in 10% ethylenediaminetetraacetic acid (EDTA) solution for 4 weeks. Following complete decalcification, the specimens were embedded in paraffin and serially sectioned. Sections were stained with hematoxylin and eosin (H&E) staining (Solarbio, G1120, China) and Masson’s trichrome staining (Solarbio, G1340, China), respectively. After staining, microscopic observation and image acquisition were performed using a VS200 automatic slide scanner (Olympus, Japan).

**Microcomputer tomography (micro-CT) scanning and analysis**

The harvested bone tissue samples were fixed and preserved in 10% formalin solution. Bone tissues were scanned using a vivaCT80 micro‑CT scanner (SCANCO Medical AG, Switzerland). Scanning parameters were set as follows: 70 kV, 112 μA, exposure time 3000 ms, and resolution 6 μm. Three‑dimensional image reconstruction of the region of interest (ROI) in bone tissue was performed using CTAn analysis software (Bruker SkyScan, Belgium), and quantitative analysis of bone morphometric parameters was conducted, including bone volume/total volume ratio (BV/TV), trabecular number (Tb.N), trabecular separation (Tb.Sp), trabecular thickness (Tb.Th), and cortical thickness (Cort.Th), to evaluate bone microstructural characteristics.

**TRAP Staining**

For cell samples, BMDMs were seeded into 24‑well plates at a density of 2×10⁵ cells per well, followed by treatment and osteoclast induction under different conditions. At the predetermined time point, the cell culture medium in each group was removed, and the cells were washed twice with pre‑cooled PBS (5 min each time), fixed with 4% paraformaldehyde at room temperature for 15 min, and washed three times with PBS (5 min each time). Staining working solution was added according to the instructions of the TRAP staining kit (Servicebio, China). After incubation at 37 °C in the dark for 60 min, the staining was terminated by washing with PBS, and observation and imaging were performed using an EVOS FL Auto Cell Imaging System (Thermo Fisher, USA).

After deparaffinization with xylene and rehydration through graded ethanol, paraffin‑embedded bone sections were incubated with staining working solution at 37 °C in the dark for 60 min according to the manufacturer’s instructions of the TRAP staining kit (Servicebio, China), followed by washing three times with PBS. After dehydration in graded ethanol, clearing in xylene, and mounting with neutral balsam, the sections were air‑dried. Image acquisition was finally performed using a VS200 automatic slide scanner (Olympus, Japan).

**Detection of bone resorption pits on bovine bone slices by scanning electron microscopy**

Bovine bone slices were sterilized in 75% ethanol, followed by washing with sterile normal saline. BMMs were seeded on bone slices and stimulated with RANKL and M-CSF to induce osteoclast formation with different treatments for 12 days. After incubation, the cells on the bone surface were thoroughly removed by ultrasonication and 1% ammonia water. Subsequently, the bone slices were dehydrated, dried, and sputter-coated with gold–palladium. The osteoclastic resorption pits on bone slices were finally observed and imaged using a scanning electron microscope (SEM).

**Immunohistochemistry staining**

Paraffin‑embedded bone sections were subjected to deparaffinization and rehydration, followed by antigen retrieval. Tissues were blocked in 5% goat serum containing 0.1% Triton X‑100 and 3% hydrogen peroxide (H₂O₂) in PBS, then incubated with IL‑6 primary antibody (diluted 1:100, Abcam, ab290735, USA), 8-OHdG primary antibody (diluted 1:500, Bioss, bs-1278R, China) and CD31 primary antibody (diluted 1:2000, Proteintech, 28083-1-AP, China) overnight at 4 °C. Immunohistochemical (IHC) staining was performed using horseradish peroxidase (HRP)‑conjugated secondary antibody, followed by visualization with 3,3'‑diaminobenzidine (DAB). Images were captured using a VS200 automatic slide scanner (Olympus, Japan).

**Sample collection, RNA extraction, RNA-seq library construction and sequencing**

At 7 days after treatment, mice were euthanized using an aneuthanasia system. The target mandibular bone tissues were rapidly dissected, collected, snap-frozen in liquid nitrogen, and subsequently stored at −80°C until RNA extraction. Total RNA was extracted from the bone tissues using TRIzol reagent (Invitrogen, Cat. No. 15596026CN, USA) according to the manufacturer’s instructions. All procedures were performed under low-temperature conditions to minimize RNA degradation.

Total RNA quality was assessed according to the RNA quality control report. Messenger RNA was enriched from qualified total RNA using oligo(dT) magnetic beads and fragmented. First-strand cDNA was synthesized using random hexamer primers, followed by second-strand cDNA synthesis. Sequencing libraries were generated through end repair, A-tailing, adapter ligation, size selection, PCR amplification and purification. Library concentration and fragment size distribution were assessed using Qubit, quantitative PCR and an Agilent Bioanalyzer. Qualified libraries were pooled according to their effective concentration and sequenced on an Illumina platform to generate paired-end reads.

**RNA-seq data processing and analysis**

Raw FASTQ reads were filtered using fastp to remove adapter-contaminated reads, reads containing more than 10% undetermined bases, and low-quality reads in which more than 50% of bases had a Phred quality score ≤5. Q20, Q30 and GC content were calculated for the clean reads. The clean paired-end reads were aligned to the reference genome using HISAT2 v2.2.1 with the corresponding gene annotation file. Gene-level read counts were obtained using featureCounts v2.0.6. Gene expression abundance was normalized as fragments per kilobase of transcript per million mapped fragments. Differential expression analysis was performed using the raw count matrix with DESeq2 v1.42.0. Nominal P value < 0.05 were considered significantly differentially expressed. DM-associated differentially expressed genes that were also significantly altered in CM/LDH versus DM were identified as DM-associated genes affected by CM/LDH treatment and visualized using VennDiagram (Version 1.8.2). Gene Ontology Biological Process and Kyoto Encyclopedia of Genes and Genomes pathway enrichment analyses were performed using clusterProfiler (Version 4.20.0). Gene set enrichment analysis was conducted using ranked gene lists and gene sets downloaded from the MSigDB website (https://www.gsea-msigdb.org/gsea/msigdb). Inflammatory and immune-related pathway activities were evaluated by single-sample gene set enrichment analysis using GSVA (Version 2.6.2), followed by min–max scaling for visualization. The SenMayo senescence-associated gene signature was used to evaluate senescence-related transcriptional changes. Representative senescence-associated secretory phenotype-related genes were selected from previously reported senescence-associated factors and plotted to compare their expression patterns among groups. Heatmaps were generated using TBtools (Version 2.475), and other plots were generated using ggplot2 (Version 4.0.3).

**Histological evaluation of major organs after CM/LDH implantation**

After establishing mandibular bone defect models in wild-type C57BL/6J mice (6 weeks old, male), GelMa hydrogel loaded with CM/LDH was implanted. After the full treatment and observation period, the mice were euthanized, and major organs (heart, liver, spleen, lung, and kidney) were collected. Major-organ histology after CM/LDH implantation was assessed by H&E staining.

**Statistical analysis**

All experiments were independently repeated at least three times unless otherwise stated. Quantitative data are presented as the mean ± SD. Statistical analyses were performed using GraphPad Prism 9.0. For comparisons among multiple groups, one-way ANOVA followed by Tukey’s multiple-comparison test was used. For comparisons between two groups, a two-tailed unpaired Student’s t-test was used when appropriate. For RNA-seq analysis, adjusted P values calculated using the Benjamini–Hochberg method were used to determine statistical significance. Detailed information on sample size, independent replicates, and statistical tests is provided in the corresponding figure legends. P < 0.05 was considered statistically significant unless otherwise specified.

**Figure S1.** High-angle annular dark-field (HAADF) and elemental mapping images of Ce-MOF and CaAl-LDH.

**Figure S2.** Hydrodynamic diameters of the Ce-MOF, CaAl-LDH and CM/LDH.

**Figure S3.** TG curves of CaAl-LDH, Ce-MOF and CM/LDH recorded under air atmosphere from 30 to 800 ℃ at a heating rate of 10 ℃ min⁻¹.

**Figure S4.** XPS spectra of Ce-MOF, CaAl-LDH and CM/LDH. (a) XPS survey spectrum (i) and high-resolution XPS spectra showing elemental analysis of Carbon (ii) and Oxygen (iii) in Ce-MOF. (b) XPS survey spectrum (i) and high-resolution XPS spectra showing elemental analysis of Oxygen (ii) and Aluminum (iii) in CaAl-LDH. (c) High-resolution XPS spectra showing elemental analysis of Carbon (i), Oxygen (ii) and Aluminum (iii) in CM/LDH.

**Figure S5.** Representative digital images and their absorbance values (at 415 nm) as a presentation for H_2_O_2_ clearance from materials at different pH values.

**Figure S6.** The Ce³⁺/Ce⁴⁺ ratios of Ce‑MOF and CM/LDH under different conditions.

**Figure S7.** The electrochemical tests of Ce‑MOF and CM/LDH. (a) The Nyquist plots of Ce-MOF and CM/LDH. (b) Cyclic voltammetry curves of Ce-MOF and CM/LDH obtained at scan rates from 10 to 200 mV/s.

**Figure S8.** Calcium ion and cerium ion release proﬁles of CM/LDH, measured in neutral solutions (pH 7.4) and acidic solutions (pH 5.0).

**Table S1.** Al ion release from CM/LDH under physiological and acidic conditions.

| Sample | 0.75 h | 3h | 6h | 12h | 24h | 48h | 72h |
| --- | --- | --- | --- | --- | --- | --- | --- |
|  | (mg L^-1^) | (mg L^-1^) | (mg L^-1^) | (mg L^-1^) | (mg L^-1^) | (mg L^-1^) | (mg L^-1^) |
| pH 5.0 | ＜0.0001 | 0.256 | 0.864 | 1.045 | 2.766 | 2.973 | 5.965 |
|  | ＜0.0001 | 0.254 | 0.886 | 1 | 2.734 | 2.947 | 5.978 |
|  | ＜0.0001 | 0.252 | 0.87 | 1.019 | 2.707 | 2.966 | 5.958 |
| pH 7.4 | ＜0.0001 | ＜0.0001 | ＜0.0001 | ＜0.0001 | ＜0.0001 | 0.003 | 0.122 |
|  | ＜0.0001 | ＜0.0001 | ＜0.0001 | ＜0.0001 | ＜0.0001 | 0.002 | 0.119 |
|  | ＜0.0001 | ＜0.0001 | ＜0.0001 | ＜0.0001 | ＜0.0001 | 0.003 | 0.123 |

**Figure S9.** Cytocompatibility and intracellular ROS-scavenging activity of CM/LDH. (a) Cell viability of BMSCs after incubation with different concentrations of Ce-MOF, CaAl-LDH, and CM/LDH for 1, 3, and 7 days, as determined by the CCK-8 assay. (b) Representative live/dead staining images of BMSCs treated with different samples at concentrations of 250 μg mL⁻¹. (c, d) Representative DCFH-DA staining images and relative fluorescence intensity quantitative analysis of intracellular ROS levels in RAW264.7 cells after different treatments under H₂O₂-induced oxidative stress. Data in bar plots are presented as the mean ± SD. n ≥ 3 biologically independent samples. Statistical significance was determined by one-way ANOVA analysis.

**Figure S10.** Representative SEM images of resorption pits formed by osteoclasts in different groups.

**Figure S11.** Representative TRAP staining images of osteoclasts with CM/LDH and acidic medium treatments.

**Figure S12.** Representative DCFH-DA staining images and MitoSOX staining images in BMDMs after CM/LDH treatments or Ce-MOF + CaAl-LDH treatment under H₂O₂-induced oxidative stress.

**Figure S13.** Representative ALP and ARS staining images of BMSCs with different treatments.

**Figure S14.** Representative EdU staining images of BMSCs under different interventions.

**Figure S15.** Representative F-actin fluorescence staining images of BMSCs under different treatment conditions.

**Figure S16.** Representative MitoSOX fluorescence staining images of BMDMs under different treatment conditions.

**Figure S17.** Representative 3D reconstructed fluorescence images of MitoTracker and mtDNA staining in BMDMs from different groups.

**Figure S18.** Representative JC-1 staining images of BMDMs from different groups.

**Figure S19.** Frequency-dependent viscoelastic properties of GelMA and GelMA + CM/LDH.

**Figure S20.** Representative DCFH‑DA staining images of H₂O₂‑stimulated RAW264.7 macrophages after treatment with GelMA and GelMA + CM/LDH.

**Figure S21.** Representative immunohistochemical staining of 8-OHdG in mandibular bone defect regions.

**Figure S22.** Representative immunohistochemical staining of CD31 in mandibular bone defect regions.

**Figure S23.** Representative images of H&E staining of the heart, liver, spleen, lung, and kidney at 4 weeks after CM/LDH administration in mandibular bone defects of wild-type mice.

**Figure S24.** Volcano plot of gene expression in DM vs Ctrl, CM/LDH vs Ctrl, and CM/LDH vs DM.

**Figure S25.** GSEA of mitochondria-related pathways in the tissue of bone defects: CM/LDH versus DM.

**References**

1. Z. Chen, Z. Li, N. Tang, et al., "Engineering Ultra‐Small Cerium‐Based Metal–Organic Frameworks Nanozymes for Efficient Antioxidative Treatment of Dry Eye Disease, " *Advanced Functional Materials* 34, no. 6 (2023), https://doi.org/10.1002/adfm.202307569.

2. H. Zhu, Z.-K. Guo, X.-X. Jiang, et al., "A protocol for isolation and culture of mesenchymal stem cells from mouse compact bone, " *Nature Protocols* 5, no. 3 (2010): 550-560, https://doi.org/10.1038/nprot.2009.238.

3. X. H. Zheng, X. Y. Zhu, X. Chen, et al., "Acsl1-Mediated Fatty Acid Synthesis Impairs Osseointegration in Type 1 Diabetes, " *Journal of Dental Research*, no. (2026), https://doi.org/10.1177/00220345261430286.

4. Y. Xiao, M. Xu, Y. Shi, et al., "ZnCe-LDO Nanozyme-Based Multifunctional Hydrogel Promotes Bone Regeneration by Inflammatory Macrophage Reprogramming and Piezo1 Activation, " *ACS Nano* 19, no. 36 (2025): 32606-32628, https://doi.org/10.1021/acsnano.5c10123.

5. G.-J. Tai, Y.-J. Ma, J.-L. Feng, et al., "NLRP3 inflammasome-mediated premature immunosenescence drives diabetic vascular aging dependent on the induction of perivascular adipose tissue dysfunction, " *Cardiovascular Research* 121, no. 1 (2025): 77-96, https://doi.org/10.1093/cvr/cvae079.

6. J. Wang, C. Zhang, W. Zhang, et al., "Ultrathin WOx Nanoribbons with Moderate ROS Clearance and Antibacterial Abilities Efficiently Induce M2 Macrophage Polarization for Diabetic Bone Defect Repair, " *Advanced Functional Materials* 34, no. 8 (2023), https://doi.org/10.1002/adfm.202306319.
